# Angiogenic and pleiotropic effects of VEGF165 and HGF combined gene therapy in a rat model of myocardial infarction

**DOI:** 10.1371/journal.pone.0197566

**Published:** 2018-05-22

**Authors:** Pavel I. Makarevich, Konstantin V. Dergilev, Zoya I. Tsokolaeva, Maria A. Boldyreva, Evgeniy K. Shevchenko, Evgeny V. Gluhanyuk, Julia O. Gallinger, Mikhail Yu. Menshikov, Yelena V. Parfyonova

**Affiliations:** 1 Laboratory of Angiogenesis, National Medical Research Center of Cardiology, Moscow, Russia; 2 Laboratory of Gene and Cell Therapy, Institute of Regenerative Medicine, Lomonosov Moscow State University, Moscow, Russia; 3 Laboratory of Gene and Cell Technology, Faculty of Medicine, Lomonosov Moscow State University, Moscow, Russia; IRCCS San Raffaele Pisana, ITALY

## Abstract

Since development of plasmid gene therapy for therapeutic angiogenesis by J. Isner this approach was an attractive option for ischemic diseases affecting large cohorts of patients. However, first placebo-controlled clinical trials showed its limited efficacy questioning further advance to practice. Thus, combined methods using delivery of several angiogenic factors got into spotlight as a way to improve outcomes. This study provides experimental proof of concept for a combined approach using simultaneous delivery of VEGF165 and HGF genes to alleviate consequences of myocardial infarction (MI). However, recent studies suggested that angiogenic growth factors have pleiotropic effects that may contribute to outcome so we expanded focus of our work to investigate potential mechanisms underlying action of VEGF165, HGF and their combination in MI. Briefly, Wistar rats underwent coronary artery ligation followed by injection of plasmid bearing VEGF165 or HGF or mixture of these. Histological assessment showed decreased size of post-MI fibrosis in both—VEGF165- or HGF-treated animals yet most prominent reduction of collagen deposition was observed in VEGF165+HGF group. Combined delivery group rats were the only to show significant increase of left ventricle (LV) wall thickness. We also found dilatation index improved in HGF or VEGF165+HGF treated animals. These effects were partially supported by our findings of c-kit+ cardiac stem cell number increase in all treated animals compared to negative control. Sporadic Ki-67+ mature cardiomyocytes were found in peri-infarct area throughout study groups with comparable effects of VEGF165, HGF and their combination. Assessment of vascular density in peri-infarct area showed efficacy of both–VEGF165 and HGF while combination of growth factors showed maximum increase of CD31+ capillary density. To our surprise arteriogenic response was limited in HGF-treated animals while VEGF165 showed potent positive influence on a-SMA+ blood vessel density. The latter hinted to evaluate infiltration of monocytes as they are known to modulate arteriogenic response in myocardium. We found that monocyte infiltration was driven by VEGF165 and reduced by HGF resulting in alleviation of VEGF-stimulated monocyte taxis after combined delivery of these 2 factors. Changes of monocyte infiltration were concordant with a-SMA+ arteriole density so we tested influence of VEGF165 or HGF on endothelial cells (EC) that mediate angiogenesis and inflammatory response. In a series of *in vitro* experiments we found that VEGF165 and HGF regulate production of inflammatory chemokines by human EC. In particular MCP-1 levels changed after treatment by recombinant VEGF, HGF or their combination and were concordant with NF-κB activation and monocyte infiltration in corresponding groups *in vivo*. We also found that both–VEGF165 and HGF upregulated IL-8 production by EC while their combination showed additive type of response reaching peak values. These changes were HIF-2 dependent and siRNA-mediated knockdown of HIF-2α abolished effects of VEGF165 and HGF on IL-8 production. To conclude, our study supports combined gene therapy by VEGF165 and HGF to treat MI and highlights neglected role of pleiotropic effects of angiogenic growth factors that may define efficacy via regulation of inflammatory response and endothelial function.

## 1. Introduction

Clinical trials of gene therapy in patients with cardiovascular diseases were extensively conducted in the previous decade and in most of them local expression of angiogenic cytokines in skeletal muscle or myocardium using non-viral or viral vectors was well-tolerated, safe (including long-term evaluation), but nonetheless—of very limited efficacy in most primary end points [1, 2].

This led to a reasonable conclusion that therapy by single gene therapy drug (“monotherapy”) is confined to effects of the molecule delivered while vessel growth depends on a large repertoire of cytokines, proteases and growth factors [3]. Thus, development of “combined gene therapy” to circumvent low efficacy hurdle was initiated and experimental studies showed that delivery of two angiogenic or tissue-protective molecules is capable to substantially enhance efficacy in animal models of ischemia. Combined gene delivery of VEGF165 with angiopoietin-1 [4], bFGF [5], urokinase plasminogen activator (uPA) [6] or SDF-1α [7] showed increase of angiogenic response compared to these molecules delivered alone. We previously reported that gene therapy by combination of VEGF165 and hepatocyte growth factor (HGF) [8] resulted in enhanced effect in a mouse model of limb ischemia and addressed potent mitogenic and pro-survival effects of VEGF165+HGF in endothelium as a putative mechanism of increased blood vessel formation [8].

Besides direct influence on EC combination of VEGF165+HGF may have peculiar pleiotropic effects on inflammatory response which is a crucial driving force of tissue repair and blood vessel maturation in ischemic tissue. “Pro-inflammatory” effects of VEGF165 are widely known: it may increase vascular permeability as well as expression of adhesion molecules and inflammatory factors by EC. On the contrary to this HGF can be referred as a “tempering” growth factor which reduces abovementioned effects of VEGF165 on endothelium [9, 10] and their combination may be of specific interest in tissues where VEGF165 induces oedema and has other adverse effects. Thus, published studies suggest that VEGF165 and HGF may “counteract” in regulation of inflammatory response and endothelium activation [9].

Importance of described properties of VEGF165 and HGF “dynamic duo” [11] becomes obvious, when addressing their potential use for treatment of MI as far as its acute phase is accompanied by robust inflammatory response. Its importance has been vividly highlighted in a classical trial dating back to 1976 which showed that immunosuppression by steroid drugs in the first days after MI resulted in dramatic worsening of outcomes [12]. Furthermore, it was shown that arteriogenesis in peri-infarct zone is driven by monocytes and depletion of this cell type reduces its efficacy and induces LV remodelling due to absence of macrophage and dendritic cells to orchestrate the process [13, 14].

Thus, combined local delivery of VEGF165+HGF to stimulate angiogenic response is supported by our and other groups’ data [8], but their potential pleiotropy is a subject to investigate. This study focuses on effects of VEGF165 and HGF after combined delivery to infarcted myocardium and investigates their molecular mechanism in EC. Influence of these growth factors on endothelium is of particular interest as these cells are involved in both—angiogenesis and inflammation standing on the “crossroads” of these reactions and being the target of gene therapy [15].

## 2. Materials and methods

### Plasmid vector design and purification

Codon-optimized cDNA of human VEGF165 and HGF were cloned to a pC4W plasmid CMV-based vector, which was characterized previously [8]. Amplification of pDNA in *E*. *coli* (DH-5α strain) and purification using Qiagen Endofree Giga kit (Qiagen, USA) was followed by LAL-test, which showed all pDNA solutions to contain <10 EU/mg of DNA.

### Animal strain and ethical approval

Wistar strain male rats (225–250 g) used for animal studies were purchased from “Puschino” SPF breeding facility (Puschino, Russia). Animals were housed in National Medical Research Center of Cardiology animal unit in individual cages with free access to drinking water and rodent chow. Natural light and controlled humidity/temperature environment were supported permanently. Husbandry, study protocol and euthanasia procedures for small and medium rodents were approved by Ethical board of National Medical Research Center of Cardiology (permit # 16-10-00).

### Model of MI and intramyocardial pDNA injection

Rats were narcotized by intraperitoneal injection of Zoletil (30 mg/kg) and were connected to a ventilator support machine for the surgery. Briefly, skin was depilated, incised and IV intercostal space was carefully dissected to move the heart into the wound. After that anterior descending coronary artery was ligated by a 3–0 silk thread 3 mm distal to left atrium appendage. Heart was placed back into the chest cavity for 3–4 minutes and after infarction zone became visible plasmid solutions were injected in peripheral area of developing myocardial ischemia. pDNA was diluted in 0.9% NaCl and injected using a 29G syringe in 4 equal injections (250 μl total). The following plasmid solutions were administered:

- empty pC4W (250 μg), n = 6;
- pC4W-VEGF165 (250 μg), n = 6;
- pC4W-HGF (250 μg), n = 6;
- 1:1 mixture of pC4W-VEGF165 and pC4W-HGF (250 μg each), n = 8;

Human HGF shares >90% sequence homology with rat including domain responsible for c-met binding [16] and human VEGF165 is one amino-acid longer that rat VEGF164 and has been shown to bind rat VEGF receptors inducing angiogenesis [17]. After pDNA injections the heart was placed back to the chest cavity and wound was closed layer-by-layer using silk ligatures. Animals were supported on a ventilator machine until restoration of spontaneous respiratory function. Skin around the sutures was wiped with 70% ethanol and animals were placed in clean individual cages for recovery. During first 4 days animals received 50 mg/kg of acetaminophen to limit postoperative pain and received required wound care to avoid infection.

### Animal euthanasia and tissue harvest for histology preparations

Prior to euthanasia rats were deeply narcotized by Isoflurane inhalation in an induction chamber and 1.0 ml of 2 mM KCl solution was slowly injected to the left ventricle by transcutaneous puncture to induce cardiac arrest within 10–20 seconds. Immediately after that cervical dislocation was performed and chest cavity was dissected along midline to excise heart. After washing in PBS atria were cut off above ligature by a razor blade and ventricular portion of the heart was frozen in Tissue Tek (Sakura Finetek, the Netherlands). Parallel frozen section of 7 μm were prepared from Tissue Tek-embedded myocardium and frozen until staining.

### Human VEGF165 and HGF assay in myocardium

To evaluate production of human VEGF165 and HGF rats with induced MI received injections of pDNA as described (n = 4/group) and were sacrificed at days 3 and 7 (2 rats each day). Animal hearts were isolated, washed in PBS, infarcted anterior wall of left ventricle (LV) was excised, cut in halves and used for explant preparation or homogenization. Explants were prepared from LV samples cut into small (2–3 mm^3^) pieces as previously described by Jang and Kim [18]. Explants were cultured in 0.5 ml of DMEM/2% FBS for 72 hours prior to ELISA. Myocardium homogenates were prepared from hearts isolated at day 3 in 800 μl of protein extraction buffer (0.5 M NaCl, 20 mM Tris (pH 7.5), 1 mM EDTA) as previously described [8]. Human HGF (Invitrogen, USA) and VEGF165 Quantikine (RnD Systems, USA) ELISA kits were used for protein assay.

### Microscopy procedures

High power fluorescent and visible light microimaging of sections was performed on Zeiss Axiovert 200M fluorescent microscope with a CCD camera Axiovision 3.1 software.

### Mallory staining and fibrosis morphometry

Mallory stain Solutions A (1% acid fuchsine), B (1% phosphomolybdic acid) and C (2% orange G, 0.5% methylene blue, 2% oxalic acid) were prepared on distilled water. Fixed slides were incubated in Solution A (2 min), Solution B (4 min) and Solution C (15 min). Between dyes slides were rinsed with distilled water and after staining sections were dehydrated and mounted in xylene-based medium. Whole sections were photographed and used for morphometry in NIH ImageJ freeware (S1 Fig).

### Assessment of cardiomyocyte length

Cardiomyocyte length correlating with contraction and hypertrophy was analyzed using fluorescent microscopy in fixed cross sections stained for laminin. After incubation with anti-laminin antibodies (Cat#ab11575, Abcam, USA, 1:100, 1 h) slides were stained by secondary AlexaFluor594-conjugated antibodies (Cat#R37117, Life technologies, USA, 1:800, 40 min). Cardiomyocytes were identified by typical shape and staining of laminin adjacent to cell membrane. Longitudinal length of 50–60 randomly selected cardiomyocytes per section was measured in Meta Imaging Series software (Molecular devices, USA).

### Morphometry of myocardium sections

Myocardial infarction measurements including epicardial, endocardial infarct lengths and circumferences were traced manually. Endocardial infarction length was assessed as length of endocardial scar that included >50% of myocardium whole thickness; epicardial infarction length was assessed as length of transmural infarction region. Epicardial and endocardial infarction ratios were obtained as follows:

$$Epicardial\;infarction\;ratio\;=\;\frac{\sum epicardial\;infarction\;lengths}{\sum infarction\;circum\;ferences}$$

$$Endocardial\;infarction\;ratio\;=\;\frac{\sum endocardial\;infarction\;lengths}{\sum infarction\;circum\;ferences}$$

finally, infarction size was calculated as:

$$Infarction\;size\;=\;(\frac{epicardial\;infarction\;ratio+endocardial\;infarction\;ratio}{2})\times 100$$

*Wall thickness* was as average thickness of three equal segments of infarcted ventricular wall. To quantitate degrees of LV dilation and infarcted wall thinning *LV dilatation index* was calculated as previously described [19]:

$$LV\;dilatation\;index\;=\;(\frac{LV\;cavity\;area}{LV\;total\;area\;})\times (\frac{wall\;thickness\;of\;uninfarcted\;region\;}{wall\;thickness\;of\;risk\;region\;})$$

### Blood vessel density evaluation

Sections were fixed in ice-cold acetone for 20 min, air-dried and washed in PBS (5 min). After washing slides were blocked by 10% normal donkey serum (30 min). Antibodies were diluted in blocking solution (1% BSA in PBS) and sections were incubated with mouse anti rat CD31 antibody (1:100, 1 hr). Then slides were washed in PBS (3×5 min) and incubated in a mixture of goat anti mouse AlexaFluor^®^594-conjugated antibodies (Cat#A-11005, Life technologies, USA, 1:800, 40 min) and anti α-SMA FITC-conjugated antibodies (Cat#F3777, Sigma-Aldrich, USA, 1:50, 1 hr). At the end of incubation nuclei were stained with DAPI and sections were mounted under coverslips. Microphotographs were taken in random fields of view (FOV) located in peri-infarct area of the section. Capillary density analysis included CD31-positive structures per field of view (FOV); arteriolar counts per FOV were estimated as number of α-SMA-positive vessels with clearly visible CD31-positive inner layer. Capillary and arteriole counts were performed by two independent persons manually and pooled to obtain mean values.

### CD68 immunostaining and monocyte counts

Fixed sections of rat myocardium were blocked and stained by primary mouse anti rat CD68 monoclonal antibodies (Cat#ab31630, Abcam, UK, 1:100, 1 hr), washed and incubated with secondary HRP-conjugated antibodies from “Vectastain ABC kit Rat IgG” (Cat#PK-4002, Vector Labs, USA). After completion of visualization nuclei were stained with hematoxylin and sections were mounted under coverslips for subsequent manual count to obtain monocyte density per mm^2^ of section area.

### Cardiac stem cell visualization and counts

For identification of the cardiac stem cells fixed sections were incubated with a goat anti rat c-kit antibody (Cat#sc1494, Santa Cruz, USA; 1:100, 2 hrs), followed by secondary donkey anti-goat AlexaFluor594 antibody (Cat#A11058, Invitrogen, USA; 1:800, 1 hr). Cardiomyocytes were visualized by staining with Troponin I antibodies (Cat#sc15368, Santa Cruz, USA; 1:400, 2 hrs), followed by donkey anti-rabbit Alexa Fluor 488 antibody (Cat#A21202, Invitrogen, USA; 1:800, 1 hr), nuclei were stained by DAPI. Data was expressed as the density of cardiac cells per mm^2^ of tissue in infarct area.

### Visualization of proliferation in myocardium sections

For analysis of cardiomyocyte proliferation immunohistochemistry with rabbit monoclonal antibody against Ki-67 (Abcam, Cat#ab16667; 1:100, 1 hr at 37°C) in formalin-fixed sections were used. Immunohistochemistry was performed using ABC kit (Vector Labs, Cat#PK-6101) with DAB and hydrogen peroxide as chromogens. Antibody diluent without primary antibody was used for negative controls. Data was expressed as density of Ki-67+ cardiomyocytes per mm^2^ of peri-infarct area.

### Assessment of apoptosis prevalence in myocardium sections

Slides with fixed sections were blocked by 10% normal donkey serum (30 min). Antibodies were diluted in blocking solution (1% BSA in PBS) and sections were incubated with primary antibodies against cleaved caspase–3 (Cat#9664S, Cell signaling, USA), washed and incubated with secondary HRP-conjugated antibodies from “Vectastain ABC kit Rat IgG” (Cat#PK-6101, Vector Labs, USA). Nuclei were stained with hematoxylin and sections were mounted under coverslips prior to manual count of apoptotic cells and nuclei to obtain normalized counts of apoptotic cells per 10^5^ cells in tissue.

### Cell lines and culture reagents

Human endothelial cell (EC) line EA.hy926 (Cat#CRL-2922, ATCC, USA) was cultured in complete DMEM-GlutaMAX^™^ (Life Technologies, USA) supplemented with 10% FBS (HyClone, USA); primary HUVEC (isolated from 5 healthy donors) was cultured in EGM-2 (Lonza, Switzerland). Cells were cultured under standard conditions (5%CO_2_, 37°C) and passaged using until passage 4–5.

### Stimulation of EC by human growth factors

EA.hy926 or HUVEC were grown in 6-well plates to 80–90% confluence, washed by PBS and 2 ml of serum-free DMEM or EBM-2 was added to wells to deprive for 5 hrs. After that medium was replaced by fresh serum-free DMEM or EBM-2 and recombinant human VEGF165 (BD, USA) or HGF (BD, USA) was added to obtain final concentration of 25 ng/ml. In certain wells both growth factors were added at 25+25 ng/ml or 12.5+12.5 ng/ml to perform combined stimulation for 2, 4, 6 or 12 hrs. At each time-point cells medium samples were collected and frozen and cells were used for mRNA or protein extraction for reverse-transcription or protein electrophoresis respectively.

### Immunoblotting

HUVEC were grown on 6-well plates, stimulated by growth factors for 15 minutes and then lysed in hot Laemmli’s buffer (150 μl/well). Obtained protein extract was collected using an insulin syringe, transferred to polypropylene tubes and heated to 95°C for 5 min. Protein extracts were resolved by SDS-electrophoresis in 10% PAAG and transferred to a PVDF membrane (Millipore, USA), which was blocked and stained (1:1000, room temperature, 1 hr) by antibody against human I-κBα (Cat#I0505, Sigma-Aldrich, USA) followed by secondary HRP-conjugated antibody (Jackson Immunoresearch, USA) for 40 min. Two-component West Pico substrate system (Pierce, USA) was used for visualization. After stripping in SDS-buffer (pH = 2.0) membrane was re-stained for phosphorylated I-κBα (Cat#ab12135, Abcam, UK) as described above. Protein load was normalized by staining for human β-actin (Cat#ab6276, Abcam, UK). Fusion-SL 3500-WL imaging system (Vilber Lourmat, Germany) was used for acquisition of signal.

### Bioplex^™^ screening and ELISA of human chemokines

Medium samples collected from HUVEC were screened for growth factors and cytokines on a multiplex Bio-Plex200 System (Bio-Rad, USA) using Cytokine Human 30-Plex Panel (Cat#LHC6003, Life Technologies, USA). Specific analysis of MCP-1 and interleukin-8 in further tests was performed using OptEIA series ELISA sets (Cat#555179 and# 555244 respectively, BD, USA).

### mRNA isolation and PCR

Total RNA was isolated using RNeasy Mini Kit (QIAGEN, USA) and first strand cDNA was synthesized using RevertAidTM First Strand cDNA Synthesis Kit (Fermentas, Latvia). PCR was performed with SYBR Green intercalating dye (Syntol, Russia) in StepOnePlus^™^ Real-Time PCR System (Applied Biosystems, USA); primers used for PCR are listed in S1 Table. After initial denaturation (95°C, 10 min), 40 amplification cycles with annealing/elongation at 60°C, 60 sec were performed for all primer pairs. Specificity of amplification was analysed by melting stage upon PCR completion.

### siRNA-mediated silencing of HIF expression

EA.hy926 cells were grown to 60–70% confluence in 6-well plates and were transfected by SMARTpool siRNA (Dharmacon, USA) against HIF-1α (Cat#D-004018-05) or HIF-2α (Cat#D-004814-02). Total 10 pmol of siRNA per well (or 20 pmol for combined silencing test) was delivered using Lipofectamine RNAiMAX reagent (Life Technologies, USA). Control Cy3-conjugated missense siRNA (Cat#AM4621, Life Technologies, USA) was used for transfection control. Efficacy of silencing was evaluated by RT-PCR using corresponding primer pairs for HIF-1α or HIF-2α mRNA.

### Luciferase-based evaluation of HIF and IL-8 promoter activity

To evaluate activity of HIF transcription factors and interleukin-8 promoter we used luminescence-based approach with a hybrid system of firefly luciferase and *Renilla* luciferase. Plasmid pGL3PGK6TKp (pHRE-Luc) contained six copies of the HIF-binding hypoxia response element (HRE) followed by firefly luciferase cDNA. EA.hy296 cells were grown to 80% confluence and transfected by pHRE-Luc using Lipofectamine 2000 (Invitrogen, Carlsbad, CA, USA). CMV-driven *Renilla* luciferase plasmid (pRen) was co-transfected for signal normalization and control of cell survival (ratio of pHRE-Luc: pRen pDNA was 5:1). Analysis of interleukin-8 expression was performed using similar protocol with a plasmid bearing luciferase under control of full-length promoter of IL-8 gene (pro_IL8-Luc) [20]. After transfection cells were incubated overnight, washed and stimulated by recombinant VEGF165, HGF or combination as described above. At the end of stimulation cells were analyzed using Dual-Luciferase Reporter Assay Kit (Promega, Fitchburg, WI, USA). Luminescence was measured using Victor^™^ X3 Multilabel Plate Reader (Perkin-Elmer Inc., USA) and ratio of firefly/*Renilla* luciferase signals was calculated.

### Statistical analysis

Statistica 8.0 (Statsoft, USA) was used for analysis of data. Statistical significance of difference was determined using Student’s t-test or Mann-Whitney rank sum U-test depending on sample distribution profile analysed by Shapiro-Wilk test.

## 3. Results

### Expression of human VEGF165 and HGF in rat myocardium after pDNA injection

**Fig 1:**
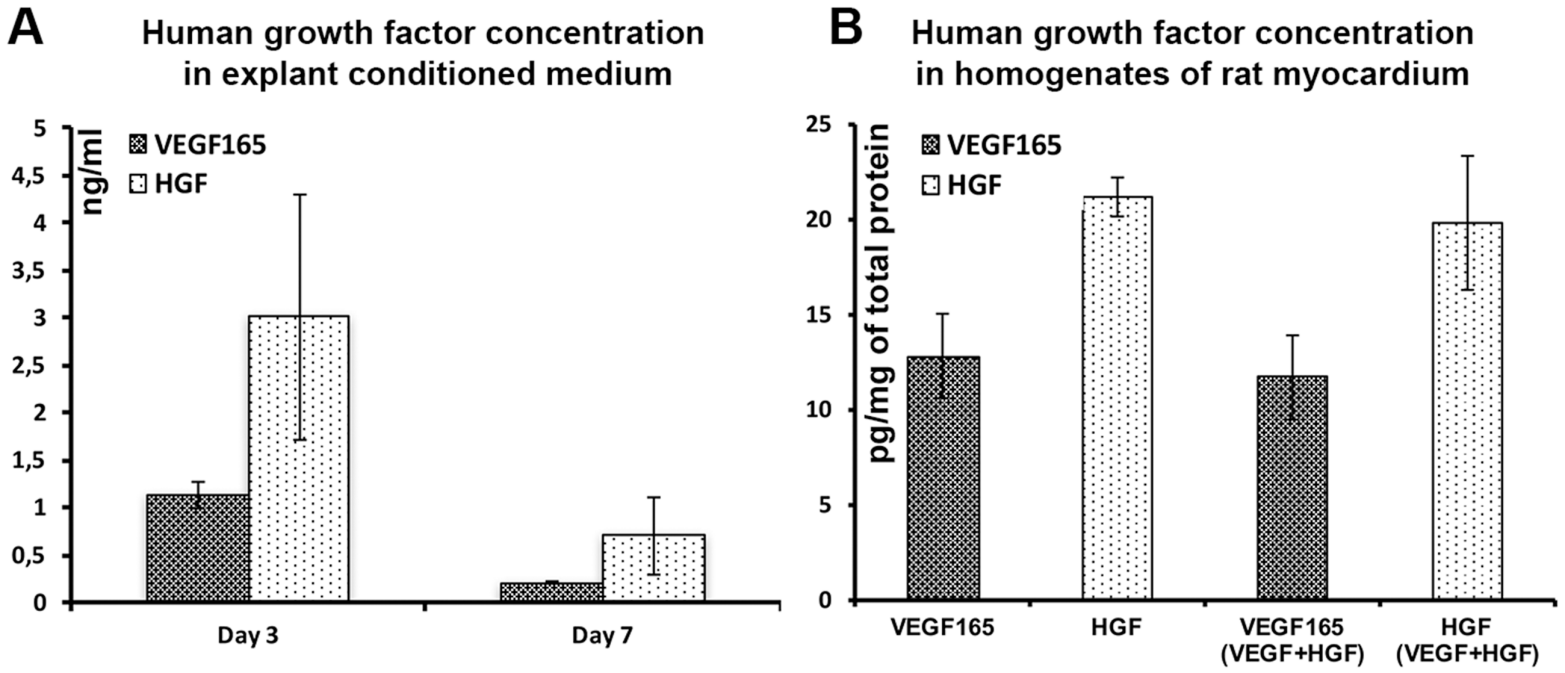
Detection of human VEGF165 and HGF in rat myocardium explants. Primary tissue was harvested at days 3 or 7 after DNA injection (A) or homogenates of left ventricle harvested at day 3 after DNA injection (B); n = 2 animals per column, ELISA data presented as Mean±S.D.

Production of human growth factors was assessed using explant culture of LV isolated at days 3 and 7 after DNA injection. Concentration of VEGF165 or HGF was assayed in explant culture medium after 72 hrs and accumulated amount of human growth factors reached order of nanogram/ml (Fig 1A). We also observed certain decline of protein production by samples isolated at 7 days compared to 3 days indicating typical dynamic of plasmid-based expression in mammalian tissues (Fig 1A).

*In situ* concentrations of VEGF165 and HGF were assessed at day 3 after injection in LV homogenates. At this point up to 12–22 pg/mg of total protein accounted for human VEGF165 or HGF. After delivery of a mixture of plasmids (Fig 1B; “VEGF+HGF” bars) content of human proteins were similar to single plasmid injection (Fig 1B; “VEGF165” and “HGF” bars) indicating comparable production of proteins after gene combined delivery.

### Combined gene therapy by VEGF165 and HGF alleviates infarction size, increases LV wall thickness and improves dilatation index

**Fig 2:**
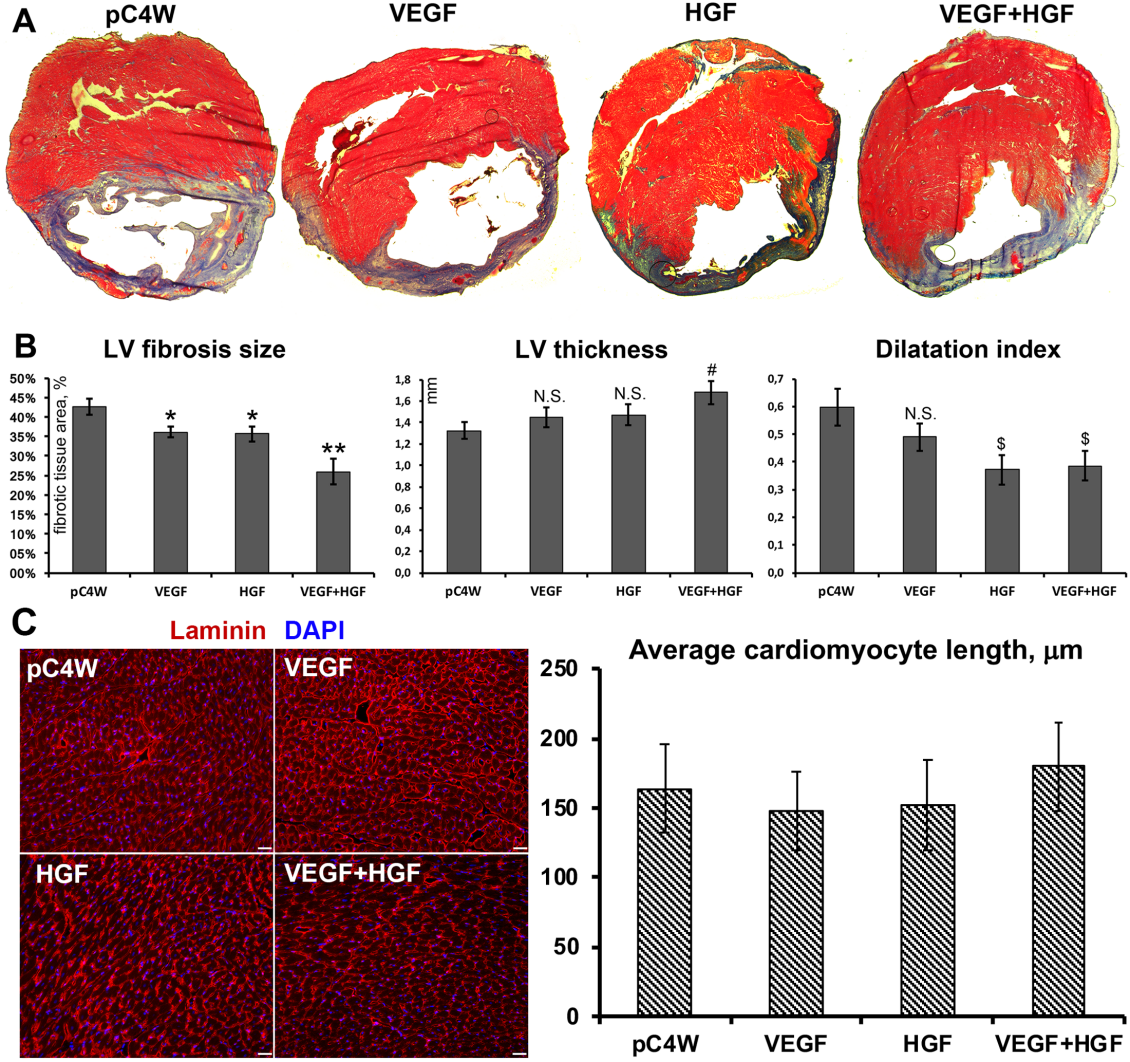
Morphometry of LV sections obtained from study groups. A—Representative images of stained sections of myocardium obtained at day 14 of experiment (fibrotic depositions stain blue); B—Results of morphometry in experimental groups treated by VEGF165, HGF or combined gene delivery; Student’s t-test (n = 4-5/group, presented as Mean±S.E.M.). LV fibrosis size graph: *p<0.05 vs “pC4W” negative control; **p<0.05 vs. “pC4W” negative control and vs. “VEGF” or “HGF” group. LV thickness graph: N.S.–not significant vs. “pC4W”; # p<0.05 vs. “pC4W”. Dilatation index graph: N.S.–not significant vs. “pC4W”; $ p<0.05 vs. “pC4W”. C—Representative images of myocardium cross-section stained for laminin (left panel) and results of cardiomyocyte length analysis in respective study groups (right graph). No significant intergroup variability was found (p>0.05).

In Mallory-stained sections of isolated hearts ventricular portion using morphometry we evaluated outcomes of therapeutic intervention at day 14 (Fig 2). Detailed description of procedure is provided in Materials and methods and S2 Fig.

In negative control animals that received empty plasmid (“pC4W” group) area of post-infarction fibrosis accounted for a total of 42.6±2.1% of LV. Injection of 250 μg of VEGF165 or HGF pDNA resulted in significant reduction of fibrosis with 36.1±1.4% (p<0.01 vs. “pC4W”) and 35.7±1.9% (p<0.05 vs. “pC4W”) in “VEGF” and “HGF” groups respectively (Fig 2B, left graph). Combined VEGF165 and HGF delivery led to significant improvement compared to each pDNA alone showing group average of 25.9±3.4% (p<0.01 vs. “pC4W” and p<0.025 vs. “VEGF” and “HGF” groups).

As for LV thickness we found it to be significantly (p = 0.01 vs. “pC4W”) increased only in animals that received combination of plasmids while delivery of each growth factor alone showed a trend to increase compared to “pC4W” negative control (Fig 2B, middle graph) without statistical significance.

Dilatation index was calculated as a quantitative value to connect LV dilation and wall thinning. Its significant reduction indicating improvement of LV geometry was observed only in HGF-treated animals (Fig 2B, right graph) including “VEGF+HGF” group while delivery of VEGF165 showed dilatation index comparable to negative control.

To avoid potential influence of cardiomyocyte contraction on morphometry results we performed staining of sections for laminin to visualize cardiomyocyte borders (Fig 2C, left panel). Measurement of longitudinal size of fibers showed no significant difference in contraction status between study groups (Fig 2C, right graph) supporting correctness of morphometry conclusions.

### Delivery of VEGF165 and HGF results in increased c-kit+ CSC count and triggers sporadic cardiomyocyte proliferation

**Fig 3:**
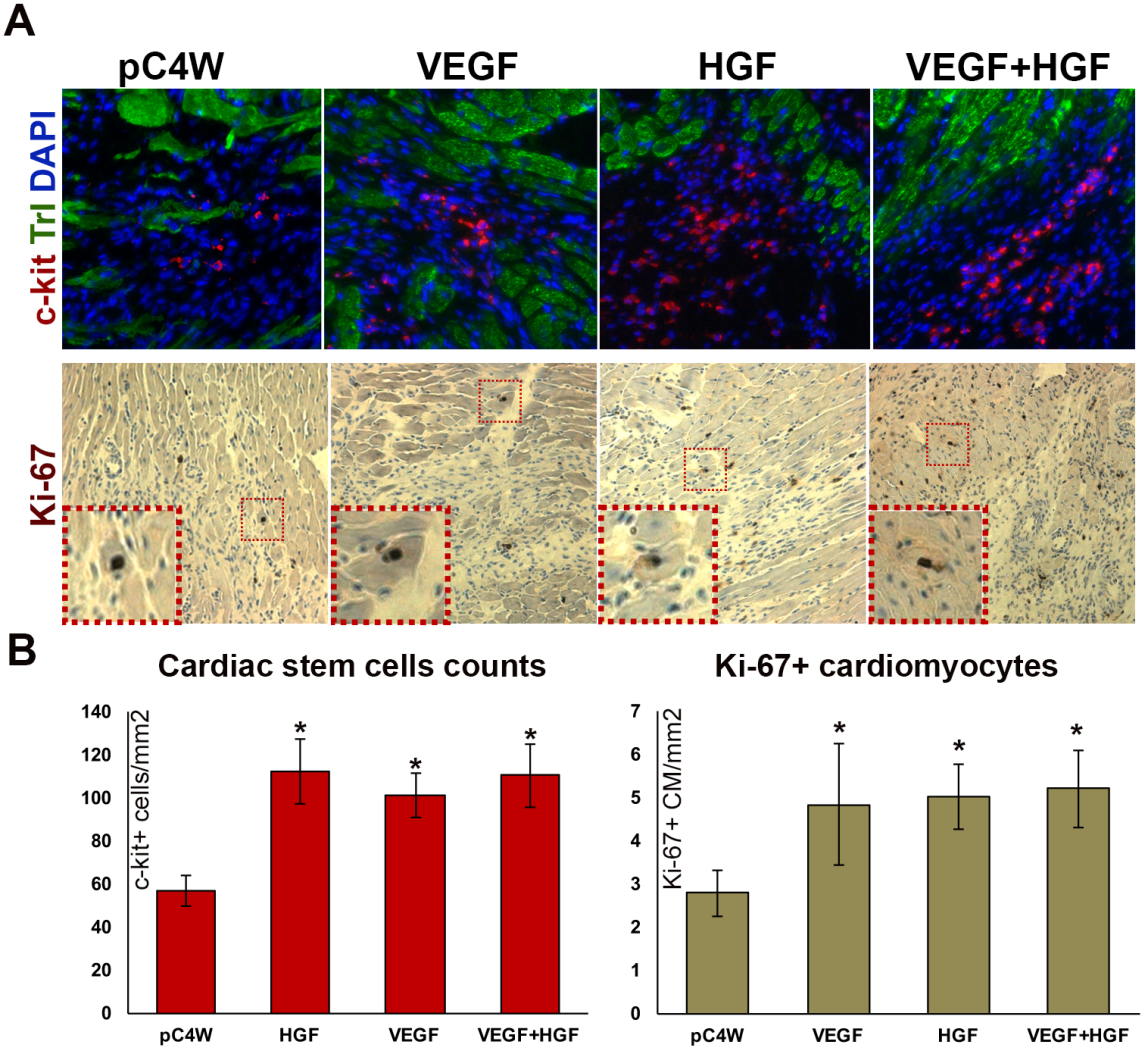
Quantitative analysis of c-kit+ CSC and Ki-67+ cardiomyocyte density. (A) Representative images of sections co-stained for c-kit/troponin-I (upper panel) and Ki-67 (lower panel); (B) Statistical analysis of c-kit+ CSC and Ki-67+ cardiomyocyte counts. n = 4-5/group, data presented as Mean±S.D.; *p<0.05 vs “pC4W” negative control (Mann-Whitney’s U-test). No significant differences were found in comparison of “VEGF+HGF” vs. “VEGF” or “HGF” groups.

Both cytokines delivered in our study have mitogenic effect on numerous cell types and play a role in mobilization or tissue-specific stem cells. Thus, we evaluated influence of gene delivery on crucial effectors of heart repair: c-kit+ cardiac stem cells (CSC) and proliferation of mature cardiomyocytes detected by Ki-67 staining (Fig 3B).

Manual counts of c-kit+ CSC showed their increased density in all treated groups, however, no additive effect was found in combined delivery group (“VEGF+HGF”) by day 14. Co-staining for troponin I allowed to distinguish between mature cardiomyocytes and CSC and visualize the latter as clusters of small c-kit+/troponin I—cells within infarct area of LV (Fig 3A).

Cardiomyocytes with Ki-67+ nuclei were sporadic throughout analyzed samples with density of 3–6 cells/mm^2^ of tissue (Fig 3A, lower panel) yet we found statistically significant increase of their counts in all treated groups. Similar to CSC we failed to show increase of proliferating cardiomyocyte counts after combining VEGF165 and HGF gene delivery (Fig 3B).

### Analysis of apoptotic cells prevalence in infarcted myocardium after gene delivery

**Fig 4:**
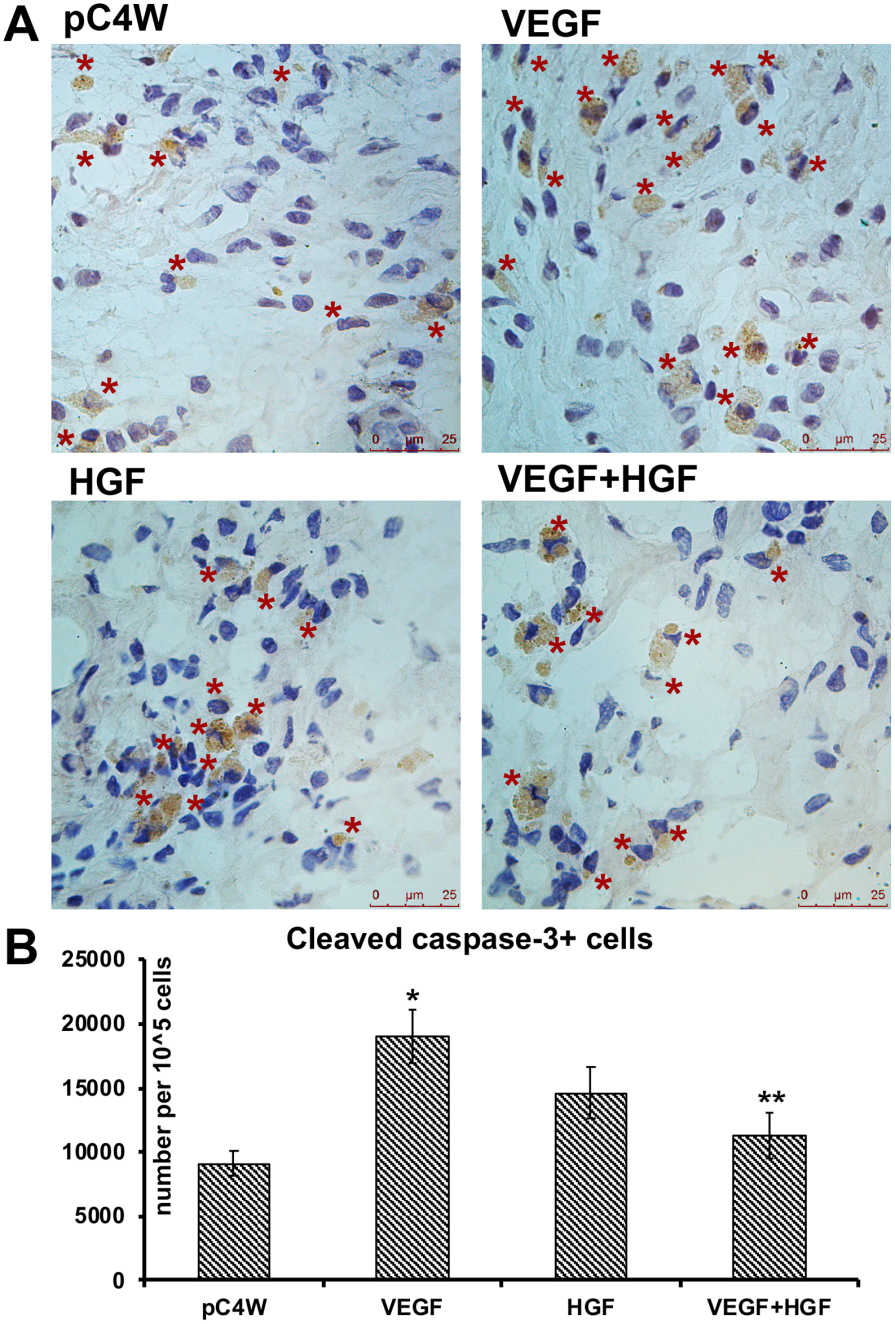
Influence of VEGF165 and HGF gene delivery on apoptosis in myocardial infarction area. A—photomicrographs of immunohistochemical visualization of cleaved caspase-3 in apoptotic cells in high-power (×630 magnification). B—Results of quantitative evaluation showing changes of apoptotic cell density (data presented as mean ±S,D.); Student’s t-test: *significant increase of apoptotic cells density (p<0.05 vs. “pC4W”); **significant decrease of apoptotic cells density (p<0.05 vs. “VEGF”).

To quantify apoptotic cells prevalence in myocardium sections we used cleaved caspase-3 immunostaining and normalized apoptotic cells number per 10^5^ cells obtained by nuclei counts in FOV. We found only sporadic cells positive for cleaved caspase-3 in remote regions of myocardium supporting local character of damage in our model. Assessment of apoptotic cell count in infarction and peri-infarct area at days 3 and 7 showed no significant differences between groups (data not shown). Interestingly, significant difference was obtained only at day 14 in infracted area where VEGF-treated group had the highest prevalence of apoptotic cells (Fig 4) with a group mean of 19010±2850 per 10^5^ cells which was significantly higher compared to control with a value of 9121±1650 per 10^5^ cells (p = 0.03). In HGF group mean value was 14613±2640 (p = 0.08 vs. pC4W and p = 0.1 vs. VEGF) and VEGF+HGF showed 11260±1920 apoptotic cells per 10^5^ cells which was significantly lower than on VEGF-treated animals (p = 0.02).

### Influence of combined VEGF165 and HGF delivery on myocardial angio- and arteriogenesis

**Fig 5:**
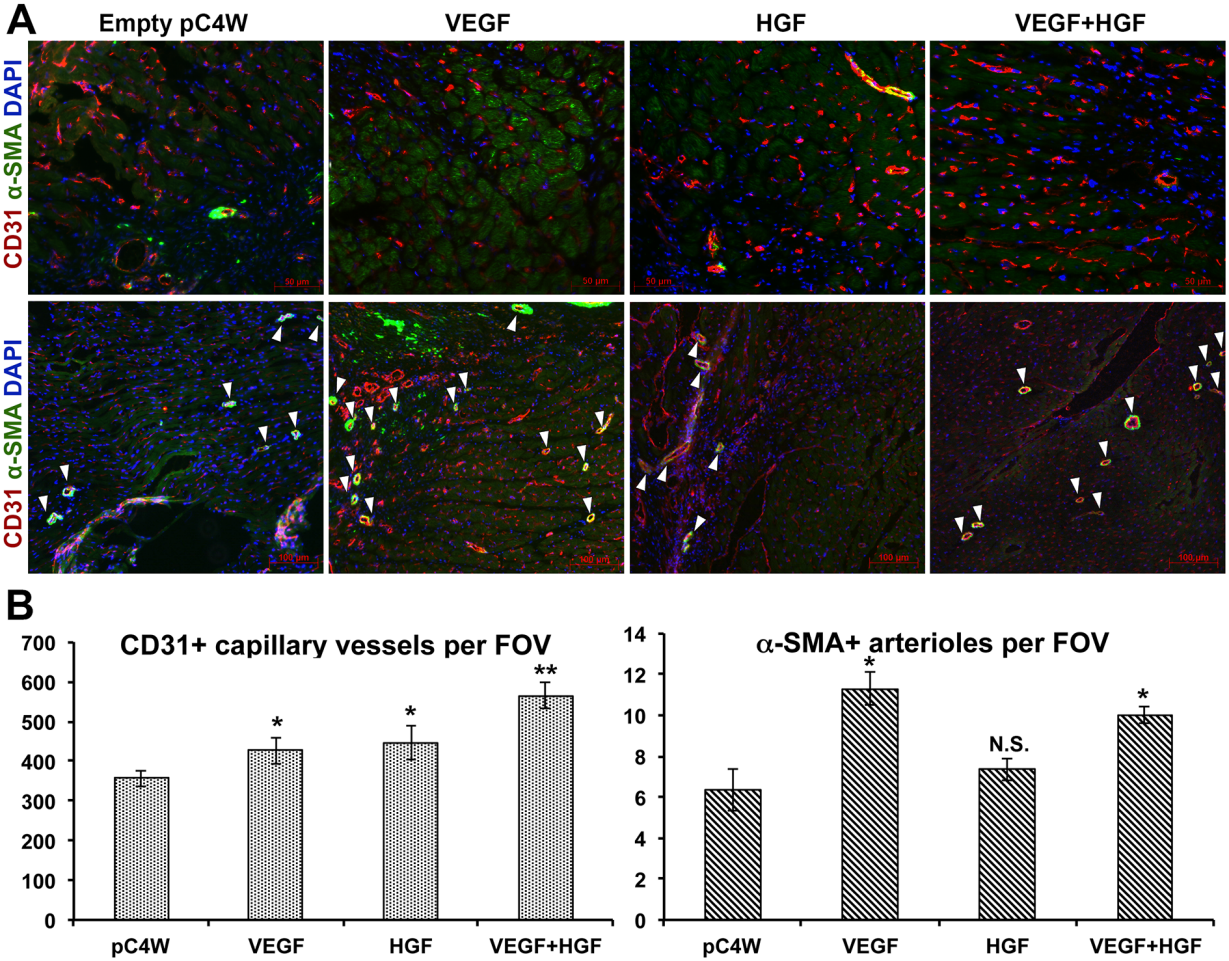
Evaluation of angio- and arteriogenesis after gene delivery of VEGF165 and HGF. A—Representative images of peri-infacrt area in cross section of myocardium after immunofluorescent visualization of CD31 and α-SMA (upper row of images in ×200 magnification emphasizes capillary morphology, lower row in ×100 magnification shows representative examples of arteriole-sized vessels); B—Results of vascular density analysis in groups treated by VEGF165, HGF or combined gene delivery (data presented as Mean±S.E.M.). Mann-Whitney U-test (n = 4-5/group): *p<0.05 vs. “pC4W” negative control; **p<0.01 vs. “pC4W”, “HGF” or “VEGF+HGF” groups; N.S.–non-significant (p>0.05 vs. “pC4W” negative control).

After injection of VEGF165 or HGF plasmid capillary density showed similar increase at day 14 compared to empty vector group (Fig 5B; left graph). Combined gene delivery group (“VEGF + HGF”) showed significantly higher capillary density than “VEGF” or “HGF” groups where each pDNA was injected alone.

Counts of α-SMA+ blood vessels indicated that arteriogenesis was effectively induced by VEGF165 while HGF failed to render impact and arteriole density in this group was comparable to “pC4W” negative control (Fig 5B; right graph). In “VEGF+HGF” group α-SMA+ arteriole counts were significantly higher than in negative control yet comparable to “VEGF”.

This set of data indicated enhanced angiogenesis induced by dual gene delivery of VEGF and HGF. However, arteriogenic response showed different pattern with unexpected lack of effect from HGF. Pleiotropy of these growth factors and their effects on inflammatory response hinted evaluation of monocyte infiltration known to be crucial for artery growth and remodelling [14].

### Gene delivery of VEGF165 and HGF modulates monocyte infiltration in peri-infarct area

**Fig 6:**
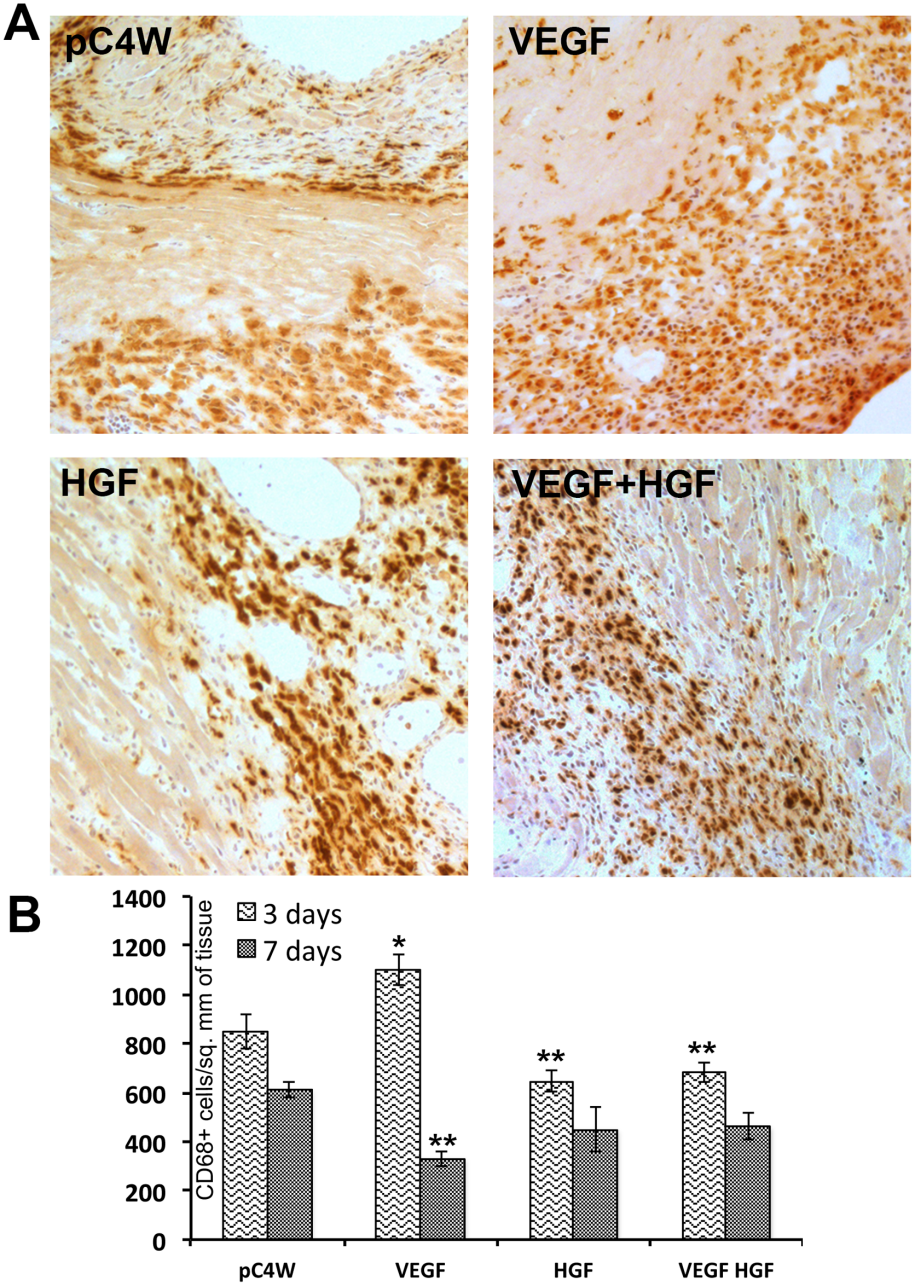
Influence of VEGF165 and HGF gene delivery on monocyte infiltration of peri-infarct area. A—Photomicrographs of immunohistochemical visualization of monocytes in myocardium samples obtained at day 3 after MI and injection of pDNA; B—Results of evaluation showing changes of CD68+ cells density per mm^2^ of tissue at days 3 and 7 of experiment (data presented as Mean±S.E.M.). Student’s t-test (n = 4/group): *significant increase of CD68+ cells density (p<0.05 vs. “pC4W” negative control); **significant decrease of CD68+ cells density (p<0.05 vs. “pC4W” at corresponding time point).

We evaluated influence of VEGF165 and HGF gene therapy on monocyte invasion due to their established role in regulation of arteriogenesis [21]. Histological assessment showed that at day 3 in negative control “pC4W” group infiltration was massive accounting >800 CD68+ monocytes/mm^2^ (Fig 6A). Delivery of VEGF165 gene resulted in increased accumulation of monocytes compared to negative control (Fig 6B; “3 days” subgroup). In HGF group this value significantly decreased compared to control and VEGF-treated animals. This indicated opposite influence of these growth factors on monocyte accumulation (Fig 6B; “3 days” subgroup). Combined delivery of VEGF165+HGF resulted in reduction of VEGF-driven monocyte infiltration and significant decrease compared to negative control. Overall, CD68+ cell counts at day 3 correlated with arteriogenic response observed in our study at Day 14 of experiment (Fig 5B; right graph).

Data at day 7 showed that in all experimental groups monocyte density was significantly reduced compared to negative control “pC4W” group (Fig 6B; “7 days” data). This pattern may be attributed to shift from acute damage to “scavenging” and repair which occurs 4–5 days after onset of ischemia [22].

### Production of MCP-1 and IL-8 by human endothelial cells treated by HGF and VEGF165

To study mechanisms underlying observed effects of VEGF and HGF delivery we focused on experiments in culture of EC. As far as our data on CD68+ monocyte accumulation at early (3 days) time point indicated differential regulation by VEGF165 and HGF we evaluated influence of these growth factors on production of chemo-, cytokines and interleukins by EC.

Multiplex assay (Bio-Plex^™^) of medium from HUVEC treated by VEGF165, HGF or both factors together showed significant changes in concentration for 3 chemokines: macrophage chemoattractant protein-1 (MCP-1), interleukin-8 (IL-8) and macrophage inflammatory protein-1β (CCL4 or MIP-1β). However, CCL4 showed very low concentrations and was not confirmed in ELISA (S2 Fig).

**Fig 7:**
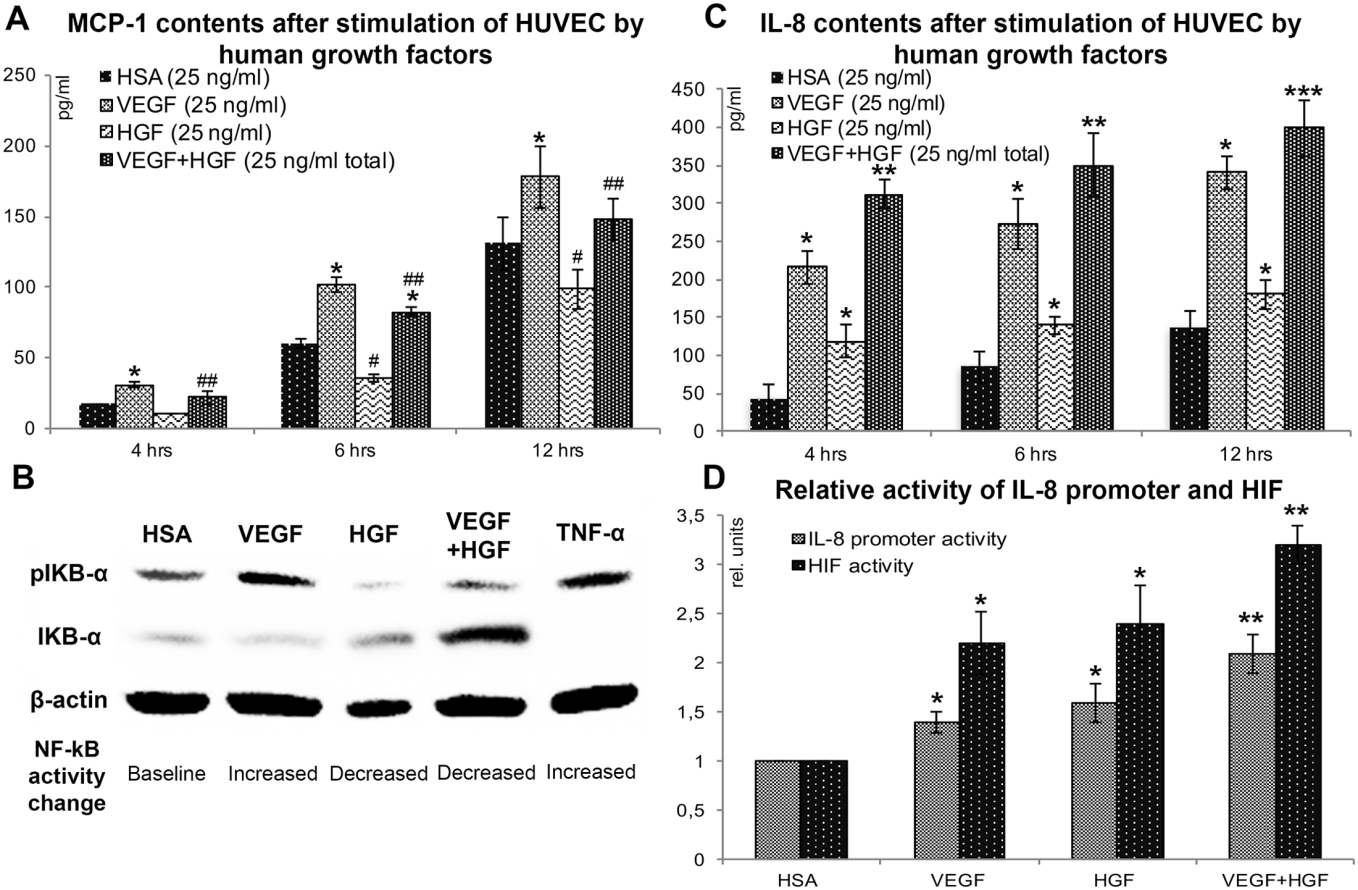
Effects of human VEGF165 and HGF on IL-8 and MCP-1 production by endothelial cells *in vitro*. A—MCP-1 production by HUVEC after stimulation with VEGF165, HGF or both factors over 4–12 hrs. Student’s t-test (Mean±S.D.; n = 4): * increased MCP-1 compared vs. HSA (p<0.05); # decreased MCP-1 compared vs. HSA or VEGF (p<0.01); ## decreased MCP-1 compared vs. VEGF (p<0.03). B—Autoradiograms of membrane stained for pIκBα и IκBα after Western blotting of extracts from HUVEC stimulated by VEGF165 (25 ng/ml), HGF (25 ng/ml) or VEGF165+HGF (25 ng/ml total); HSA (25 ng/ml) served as a negative, and TNF-α (5 ng/ml) as a positive control. C—IL-8 production by HUVEC after stimulation with VEGF165, HGF or both factors over 4–12 hrs. Student’s t-test (Mean±S.D.; n = 3–4): * increased IL-8 compared vs. HSA (p<0.05); ** increased IL-8 compared vs. VEGF or HGF (p<0.001); *** increased IL-8 compared vs. VEGF or HGF (p<0.01). D—Activation of HIF and IL-8 promoter in EA.hy926 after treatment with VEGF, HGF and their combination. Data of luciferase-based reporter assay (see Materials and methods for detailed description) Mann-Whitney U-test (Mean±S.D.; n = 3–4): *p<0.05 vs HSA; **p<0.025 vs. VEGF or HGF.

After multiplex screening we switched to protein-specific ELISA of conditioned medium from HUVEC and reproduced changes of MCP-1 and IL-8. Production of MCP-1 increased after treatment by VEGF165 and significantly dropped after addition of HGF and addition of growth factors in combination resulted in reduction of VEGF-driven increase of MCP-1 content (Fig 7A).

At the same time IL-8 increased after addition of both—VEGF165 and HGF resulting in additive rise of IL-8 concentration after co-stimulation of EC by VEGF and HGF (Fig 7C). Thus, we found that two crucial inflammatory chemokines showed differential response to HGF and—consequently—to combination of VEGF and HGF.

### Changes of NF-κB pathway activation by VEGF165 and HGF correlate with MCP-1 production by HUVEC

Expression of chemokines involved in inflammatory response is controlled by NF-κB transcription factor. After PCR experiments that showed changes of MCP-1 mRNA expression concordant with ELISA results (data not shown) we evaluated NF-κB activity using Western blotting for an inhibitory kinase IκB and its phosphorylated form to obtain pIκB/IκB ratio.

At 30 min of incubation Western blotting showed increase of pIκB and decrease of IκB in VEGF-treated HUVEC and opposite trend after addition of HGF (Fig 7B). Addition of VEGF + HGF in total concentration on 25 ng/ml (12,5 ng/ml each) resulted in reduction of VEGF-induced IκB phosphorylation. After treatment by TNF-α (5 ng/ml) as a positive control we observed maximal increase of pIκB and found no band for IκB indicating total conversion of kinase to its phosphorylated form. Thus, we found that in EC changes of MCP-1 expression induced by VEGF, HGF or their combination were concordant with activity of NF-κB controlling MCP-1 mRNA expression.

### HIF-2 is a putative regulator of IL-8 production in endothelial cells

Expression of IL-8 gene can be controlled by major HIF isoforms—HIF-1 and HIF-2 due to presence of “hypoxia-responsive element” (HRE) sequence in its promoter region [23]. We utilized luciferase-based reporter plasmids to transfect them into human EC line (EA.hy926). Using a pHRE-LUC plasmid encoding firefly luciferase and bearing 6 repeats of HRE sequences to make its expression HIF-sensitive we assayed influence of growth factor addition on HIF-activity. As for IL-8 we also used a plasmid where expression of firefly luciferase was driven by IL-8 gene promoter (pro_IL-8-LUC). Using co-transfection by these 2 plasmids to EA.hy926 we showed a strong positive (r^2^ = 0.77, p = 0.01) correlation of HIF activity and activation of IL-8 promoter region (Fig 7D). We also conducted this experiment in 2 separate cultures to obtain similar results indicating that treatment by VEGF and HGF triggers 2 concordant events—activation of HIF and IL-8 gene promoter region. Combined treatment by VEGF+HGF resulted in additive-like effects with maximum activation of HIF and IL-8 promoter. However, further evaluation was required due to non-specificity of this assay depending on activity of all known HIF isoforms known to have differential functions.

To evaluate effects of HIF-1 and HIF-2 we used specific siRNA against HIF-1α and HIF-2α subunits of corresponding proteins. Negative control missense siRNA (Cy3-siRNA) was conjugated with Cy3 fluorophore to control transfection efficacy.

**Fig 8:**
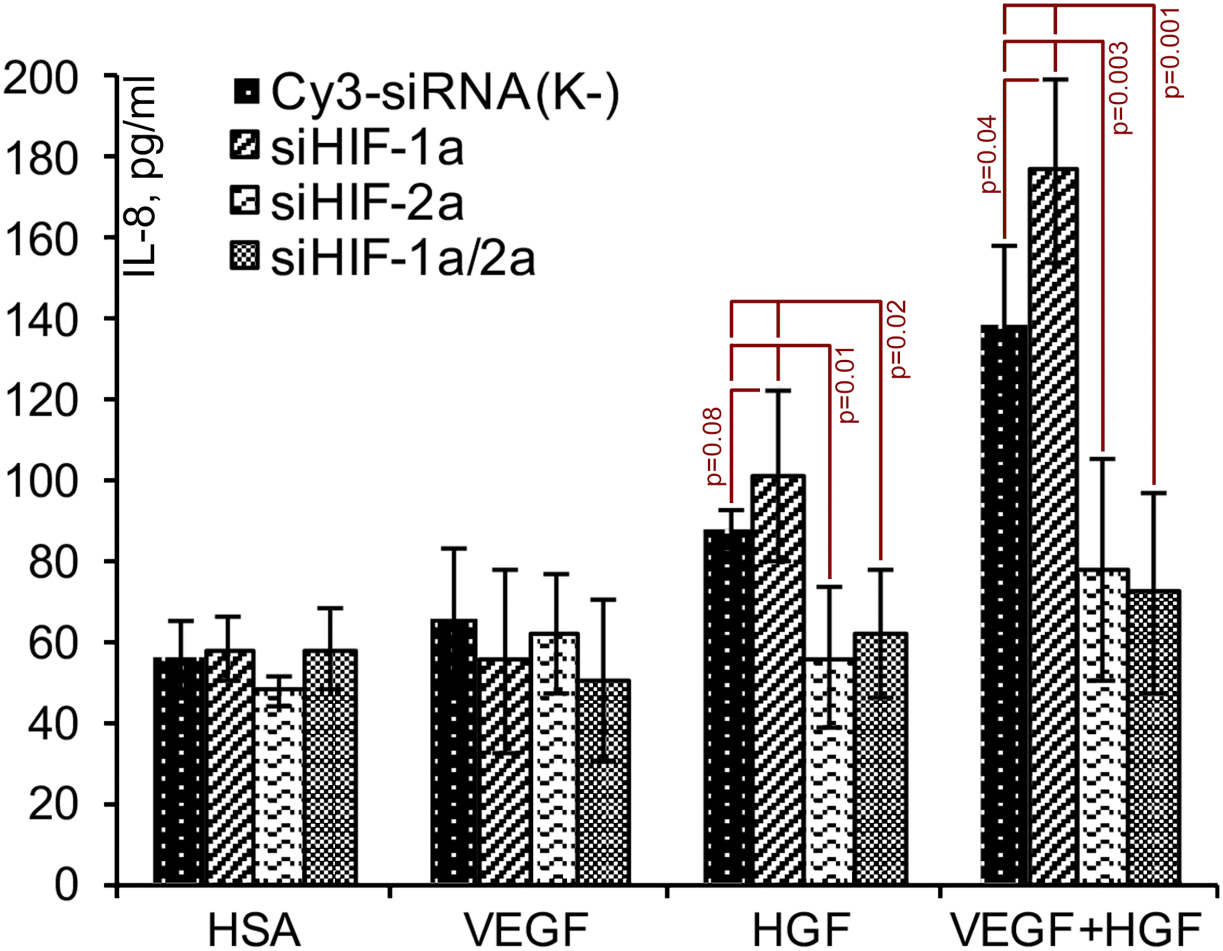
Influence of HIF-1/2α «knockdown» on IL-8 production by endothelial cells (in response to VEGF, HGF or their combination. EA.hy926 cells were transfected with respective siRNA and after 24 hrs were incubated with human serum albumin (HSA), VEGF165, HGF or their combination for 6 hrs. Student’s t-test (mean±S.D, n = 4): p-values are given at the graph.

After stimulation of cells by VEGF, HGF and their combination we found that “knockdown” of HIF-1α expression has no significant effects on IL-8 expression were observed except for VEGF+HGF (Fig 8, * mark). Under suppressed HIF-1α expression combination of VEGF+HGF gave the highest yield of IL-8 reaching 180 pg/ml. In contrast to that “knockdown” of HIF-2α or both isoforms (Fig 8, “siHIF-1α/2α” bars in “HGF” and “VEGF+HGF”) resulted in dramatic drop of IL-8 concentration in response to HGF and its combination with VEGF165 (Fig 8). this suggested potential role of HIF-2 in control of IL-8 production by EC with no obvious impact of HIF-1 isoform except for a trend to increased production of IL-8 in response to HGF (Fig 8, “HGF”; p = 0.08).

## 4. Discussion

Our previously published data showed that in animals with limb ischemia combination of VEGF and HGF-encoding pDNAs resulted in enhanced angio- and arteriogenesis in skeletal muscle compared to each factor alone [8]. In present study we shifted our focus to MI as an indication targeted in numerous trials of therapeutic angiogenesis that showed limited efficacy.

We found that injection of 250 μg of pDNA to rat myocardium resulted in expression of human VEGF165 and HGF detectable by ELISA in explant culture (accumulated production) and in tissue homogenates from site of injection (*in situ* production). Our data provided proof of approximately 1:1 production of VEGF165 and HGF after injection of a mixture of plasmids and showed that combined delivery results in local concentration similar to injection of each pDNA alone (Fig 1).

Animal test provided valuable data about early events occurring within 14 days after gene therapy during acute phase of MI. This time point is rarely considered informative in models of MI and most experiments rely on ultrasound to assess functional outcome within long-term period (2–3 months). We chose detailed morphometry to investigate changes driven by gene therapy within first weeks after MI. We also chose this duration of experiment as far as expression of protein after single injection of pDNA typically lasts 10–14 days in most mammalian tissues supporting our design as an adequate way to detect early effects of plasmid-based gene therapy [24].

Morphometry showed that both—VEGF and HGF—alleviated postinfarction fibrosis of LV (Fig 2A). Our study has certain limitations due to lack of ultrasound evaluation at a later time point (typically done at 2–3 months) yet histological data clearly indicated reduction of fibrosis which is a well-known predictor of beneficial outcome in MI. Mechanism of this effect is associated with antifibrotic effects of these growth factors and their ability to restore blood flow and tissue nutrition. Antifibrotic influence of HGF in MI is defined by its ability to reduce collagen types I and III production by cardiac fibroblasts expressing c-met in abundance [25]. It is peculiar that after onset of myocardial ischemia expression of HGF receptor (c-met) increases dramatically indicating local defensive reaction and an attempt to “sensitize” ischemic tissue to effects of HGF [26]. Importantly, we found that combined delivery group showed the lowest fibrosis area throughout the experiment (Fig 2B) with a quasi-additive type of influence. Similar data obtained in rat MI model was published by a group that used a hydrogel with controlled release of VEGF165 and HGF [27]. Another positive change in MI geometry increased thickness of LV wall yet it achieved statistical significance only in “VEGF+HGF” group providing evidence for therapeutic superiority of this combination. This point is a subject for more detailed discussion as it may involve different modes of action of delivered growth factors.

Regeneration of myocardium relies on several mechanisms including activation of resident c-kit+ cells (known as “cardiac stem cells” or CSC) and limited proliferation of cardiomyocytes [28]. We found that gene delivery of VEGF165, HGF or their combination resulted in comparable increase of CSC number in peri-infarct area and sporadic proliferation of mature cardiomyocytes (Fig 3). These effects may occur via activation of VEGFRs and c-met that launch mitogenic signaling cascades in many mammalian tissues. As for CSC mobilization and differentiation for HGF it has been shown that its exogenous expression in myocardium induces CSC proliferation and—putatively—differentiation to cardiomyocytes and EC to support heart repair [29]. It has been established that activation of Akt axis by growth factors is involved in regulation of CSC proliferation and differentiation and HGF was identified as a factor activating a chain of events leading to formation of new cardiac tissue [30]. Potentially this mechanism may contribute to significant increase of LV thickness observed in “VEGF+HGF” group (Fig 2B).

In our study increase of wall thickness, higher CSC number and cardiomyocyte proliferation were concordant with morphometry that showed alleviation of dilatation index in HGF-treated animals including “VEGF+HGF” group (Fig 2B) indicating prevention of negative LV remodeling. Similarly, in pigs with MI delivery of HGF by adenovirus has shown therapeutic potential (improved cardiac function and perfusion) and activated focal cardiomyocyte proliferation [31].

Delivery of angiogenic growth factors to peri-infarct zone is intended to induce formation of new capillaries to reduce ischemic damage at later stages of tissue repair. Our data from histology studies showed that both—VEGF165 and HGF effectively induced angiogenesis (Fig 5) and additive effect was observed in combined delivery groups. Increase of angiogenic response by addition of VEGF165 to HGF has been established in a number of models [32, 33] and our previous data [8, 34] was concordant with present study supporting common mechanism of capillary formation in human tissues [35].

However, another crucial process known as arteriogenesis defined as formation of α-SMA+ vessels via collateral remodelling showed. Here we were surprised to find lack of effect from HGF delivery and its “dampening” effect on VEGF-driven arteriogenesis clearly observed in respective group (Fig 5B). This could result from low expression of HGF in the tissue so we addressed published data. In our study tissue homogenates contained 20–25 pg of HGF per mg of total protein (Fig 1). Similar values (≈5–10 pg/mg) were published by Aoki in a study using HGF-encoding pDNA in a rat model of MI [36]. In big animal model (goat) HGF concentration was even lower (~1 pg/mg) yet sufficient to alleviate LV remodelling and induce angiogenesis [37]. Thus, we came to conclusion that chosen delivery method was performing soundly and sought for further explanation.

Among a vast variety of cells infiltrating myocardium after MI monocytes share a specific role as they give rise to macrophages [38] to scavenge necrotic tissue, orchestrate regeneration and recovery of blood supply via vessel formation. Arteriogenesis is sensitive to depletion of monocytes and depends on macrophages in many tissues including myocardium[39–41]. Thus, we suggested that pleiotropic effects of VEGF165 and HGF on monocyte recruitment could define arteriogenic response induced by these growth factors.

Another unexpected finding was increase of apoptotic cells at Day 14 in infarction area in VEGF treated group (Fig 4). This can hardly be due to immune response against transgenic human protein as apoptosis prevalence was decreased in dual gene delivery group (Fig 4B). This might have resulted from apoptosis of infiltrating cells which we clearly saw in microphotographs (Fig 4A). Indeed, most cleaved caspase-3-positive cells were small round-shaped cells residing within forming scar and possibly originating from initial infiltration. Increase of apoptosis in VEGF group was attributed to a massive infiltration observed in this group due to growth factor’s pro-inflammatory properties and increased vascular permeability—a characteristic feature of VEGF-driven angiogenesis [4, 11]. We found no difference in apoptotic cardiomyocyte counts at Day 3 and 7 which corresponds to early time points where angiogenesis is still incomplete and severe ischemia is overwhelming and pro-survival effects of express growth factors.

Indeed, changes of monocyte counts at 3 days (Fig 6) were concordant with arteriole density at 14 days (Fig 5B). Injection of pDNA with VEGF165 resulted in significant increase of monocyte infiltration reflecting “pro-inflammatory effect” described of VEGFs [42, 43]. VEGF165 may influence monocyte invasion by activation of NF-κB in EC which facilitates monocyte rolling and invasion [9]. It may also act directly via its receptors on attracted monocytes and VEGFR1 known to be a “decoy receptor” on EC is identified as an important trigger of monocyte migration and phenotype changes [44, 45]. In contrary to this expression of HGF resulted in decrease of monocyte number in peri-infarct area which was concordant with lack of arteriogenic response to HGF (Figs 5B and 6B).

We concluded that pDNA-based expression of HGF in peri-infarct area resulted in reduced monocyte taxis with subsequent lack of arteriogenic response which was in disagreement with the fact that *in vitro* HGF is a chemoattractant for epithelium, endothelium and smooth-muscle cells [46–48]. However, immature monocytes and certain macrophage subtypes are known to have a very small subpopulation of CD68+ positive cells bearing HGF receptor—c-met [49] so direct influence of HGF on these cells was unlikely to play a role. Basing on importance of endothelium as a barrier to control invasion we investigated influence of VEGF165, HGF and their combination on production of molecular messengers that control inflammation [50].

After ELISA of culture medium from EC treated by VEGF165, HGF or both factors we found significant changes of two chemokines—IL-8 and MCP-1. It has been shown that IL-8 may be a regulator of angiogenesis acting via its receptor on EC rendering angiogenic response in culture and *in vivo* [51, 52]. For MCP-1 potent involvement in arteriogenesis and collateral remodelling has been established and these effects are attributed to attraction of monocytes that secrete a “cocktail” of bioactive molecules inducing smooth muscle cells proliferation [53].

Changes of IL-8 and MCP-1 production hinted investigation into transcriptional regulators involved in control of their gene expression. We found influence of VEGF165 and HGF on NF-κB with VEGF165 increasing activity of this regulatory pathway and HGF reducing in accordance with their “pro-”and “anti-inflammatory” profiles respectively (Fig 7B). Similar has been shown by Min et al. [9] focusing mostly on leukocyte adhesion to endothelium monolayer regulated by NF-κB via expression of ICAM and VCAM. However, they did not use an *in vivo* model to show relevance of these effects or their correlation with physiological reactions. In present study we found that dynamics of change of MCP-1 was concordant with modulation of NF-κB pathway (Fig 7A and 7B). Furthermore, it resembled changes of CD68+ cells density after injection of plasmids (Fig 6) and—finally—provided a putative explanation of lack of effect for HGF on arteriogenic response (Fig 5B). Thus, we found VEGF165 to be a driver of NF-κB-dependent expression of MCP-1 while HGF seemed to be a “tempering” player reducing NF-κB activation and MCP-1 production providing ground for changes of monocyte infiltration with subsequent effect on arteriogenic response.

IL-8 is known to be regulated by NF-κB as well and its promoter area bears a sequence binding RelA subunit of NF-κB [54]. Nevertheless, in our experiments activity of NF-κB pathway was inhibited by HGF which suggested an alternative regulatory mechanism of IL-8 increase in response to HGF.

Involvement of IL-8 in angiogenesis relies on its expression in cells under hypoxic conditions which is mediated by HIF transcription factors. Besides O_2_ partial pressure HIF family is under downstream control of many tyrosine-kinase receptors including c-met and VEGFRs and their activation is known to result in stabilization of HIFs under normoxic conditions by direct phosphorylation of HIF-1α and HIF-2α subunits [55, 56]. These hypoxia-inducible subunits form heterodimeric complexes with constitutive HIF-1β subunit to form HIF-1 and HIF-2 respectively.

Quantitative analysis of HIF activity showed that VEGF165 and HGF increased it (putatively via described phosphorylation pathway) with maximum effect observed in VEGF165+HGF which correlated with IL-8 promoter activation assessed by another reporter construct in the same culture (Fig 7D).

Analysis using siRNA revealed critical role of HIF-2 in control of IL-8 expression by human endothelium (Fig 8). Knockdown of HIF-2α subunit and subsequent loss of HIF-2 activity resulted in dramatic drop of IL-8 production driven by HGF or its combination with VEGF165. Knockdown of HIF-1α seemed to slightly boost production of IL-8 triggered by HGF and VEGF+HGF which is an accordance with data on inhibitory effect of HIF-1 on IL-8 production [23]. Both—HIF-1 and HIF-2 bind HRE sequence in promoter of IL-8 yet their counterbalancing effects are defined by co-factors forming heteromeric complex with HIFs after their binding to DNA [23]. Importance of this finding is supported by the fact that IL-8 is known to have pro-angiogenic effects [57, 58] and, thus, its HIF-2-dependent expression may play a role in angiogenic effect of growth factors—namely VEGF165 and HGF [59].

## 5. Conclusions

Our study has shown that combined gene delivery of VEGF165 and HGF to peri-infarct area has significant therapeutic benefits compared to each gene alone. Combined gene therapy showed maximum efficacy in terms of MI size reduction and it was the only group where LV thickness was increased. Mechanism of action of VEGF165 and HGF gene therapy may rely on activation of CSC and cardiomyocyte proliferation and we found evidence for both processes yet further time points are to be assessed in the future. Furthermore, capillary density was increased after gene therapy by VEGF165 or HGF alone yet combined delivery resulted in maximum amount of capillaries.

We also found that pleiotropic effects of VEGF165 and HGF in infarcted myocardium may influence arteriogenic response via regulation of chemokines that control monocyte invasion. Interestingly, in our study HGF dampened effects of VEGF165 on monocyte invasion and arteriogenesis by reduction of NF-κB activity and MCP-1 production by EC. However, HGF was activating production of IL-8 by EC which was HIF-dependent with a major role of HIF-2.

To conclude, our study is not limited to evaluation of therapeutic efficacy but also highlights the role of pleiotropy established for many growth factors and cytokines. This should be taken into account during development of new methods and approaches in therapeutic angiogenesis and our case is a vivid example of such unexpected modality of action.

## Supporting information

S1 Fig Graphic description of morphometry methods used in the study. (TIF)

S2 Fig Ranking of Bioplex^™^ results after assessment of conditioned medium from HUVEC treated by VEGF165, HGF or their combination. HI—heat index, OOR—our of range, LD- limit of detection, NS—not significant difference between samples, SIG—significant difference between samples.
(TIF)

S1 Table Primers used for semi-quantitative PCR. (DOCX)

S1 File ARRIVE guidelines checklist. (PDF)

## References

[1] De Haro J, Acin F, Lopez-Quintana A, Florez A, Martinez-Aguilar E, Varela C. Meta-analysis of randomized, controlled clinical trials in angiogenesis: gene and cell therapy in peripheral arterial disease. Heart and vessels. 2009;24(5):321–8. doi: 10.1007/s00380-008-1140-z . 19784813 10.1007/s00380-008-1140-z

[2] Miao YL, Wu W, Li BW, Fang WW, Liu Y, Li L, et al Clinical effectiveness of gene therapy on critical limb ischemia: a meta-analysis of 5 randomized controlled clinical trials. Vascular and endovascular surgery. 2014;48(5–6):372–7. doi: 10.1177/1538574414539397 . 24951292 10.1177/1538574414539397

[3] Gupta R, Tongers J, Losordo DW. Human studies of angiogenic gene therapy. Circulation research. 2009;105(8):724–36. doi: 10.1161/CIRCRESAHA.109.200386 . 19815827 10.1161/CIRCRESAHA.109.200386 PMC2770893

[4] Liu X, Chen Y, Zhang F, Chen L, Ha T, Gao X, et al Synergistically therapeutic effects of VEGF165 and angiopoietin-1 on ischemic rat myocardium. Scandinavian cardiovascular journal: SCJ. 2007;41(2):95–101. doi: 10.1080/14017430701197593 . 17454834 10.1080/14017430701197593

[5] Lee JS, Kim JM, Kim KL, Jang HS, Shin IS, Jeon ES, et al Combined administration of naked DNA vectors encoding VEGF and bFGF enhances tissue perfusion and arteriogenesis in ischemic hindlimb. Biochemical and biophysical research communications. 2007;360(4):752–8. doi: 10.1016/j.bbrc.2007.06.120 . 17624309 10.1016/j.bbrc.2007.06.120

[6] Traktuev DO, Tsokolaeva ZI, Shevelev AA, Talitskiy KA, Stepanova VV, Johnstone BH, et al Urokinase gene transfer augments angiogenesis in ischemic skeletal and myocardial muscle. Molecular therapy: the journal of the American Society of Gene Therapy. 2007;15(11):1939–46. doi: 10.1038/sj.mt.6300262 . 17653104 10.1038/sj.mt.6300262

[7] Yu JX, Huang XF, Lv WM, Ye CS, Peng XZ, Zhang H, et al Combination of stromal-derived factor-1alpha and vascular endothelial growth factor gene-modified endothelial progenitor cells is more effective for ischemic neovascularization. Journal of vascular surgery. 2009;50(3):608–16. doi: 10.1016/j.jvs.2009.05.049 . 19595531 10.1016/j.jvs.2009.05.049

[8] Makarevich P, Tsokolaeva Z, Shevelev A, Rybalkin I, Shevchenko E, Beloglazova I, et al Combined transfer of human VEGF165 and HGF genes renders potent angiogenic effect in ischemic skeletal muscle. PloS one. 2012;7(6):e38776 doi: 10.1371/journal.pone.0038776 . 22719942 10.1371/journal.pone.0038776 PMC3374822

[9] Min JK, Lee YM, Kim JH, Kim YM, Kim SW, Lee SY, et al Hepatocyte growth factor suppresses vascular endothelial growth factor-induced expression of endothelial ICAM-1 and VCAM-1 by inhibiting the nuclear factor-kappaB pathway. Circulation research. 2005;96(3):300–7. doi: 10.1161/01.RES.0000155330.07887.EE . 15637298 10.1161/01.RES.0000155330.07887.EE

[10] Mizuno S, Nakamura T. Prevention of neutrophil extravasation by hepatocyte growth factor leads to attenuations of tubular apoptosis and renal dysfunction in mouse ischemic kidneys. The American journal of pathology. 2005;166(6):1895–905. doi: 10.1016/S0002-9440(10)62498-4 . 15920173 10.1016/S0002-9440(10)62498-4 PMC1602426

[11] Gerritsen ME. HGF and VEGF: a dynamic duo. Circulation research. 2005;96(3):272–3. doi: 10.1161/01.RES.0000157575.66295.e0 . 15718506 10.1161/01.RES.0000157575.66295.e0

[12] Roberts R, DeMello V, Sobel BE. Deleterious effects of methylprednisolone in patients with myocardial infarction. Circulation. 1976;53(3 Suppl):I204–6. . 1253361

[13] Moldovan NI, Goldschmidt-Clermont PJ, Parker-Thornburg J, Shapiro SD, Kolattukudy PE. Contribution of monocytes/macrophages to compensatory neovascularization: the drilling of metalloelastase-positive tunnels in ischemic myocardium. Circulation research. 2000;87(5):378–84. . 10969035 10.1161/01.res.87.5.378

[14] van Amerongen MJ, Harmsen MC, van Rooijen N, Petersen AH, van Luyn MJ. Macrophage depletion impairs wound healing and increases left ventricular remodeling after myocardial injury in mice. The American journal of pathology. 2007;170(3):818–29. doi: 10.2353/ajpath.2007.060547 . 17322368 10.2353/ajpath.2007.060547 PMC1864893

[15] Rubanyi GM. The role of endothelium in cardiovascular homeostasis and diseases. Journal of cardiovascular pharmacology. 1993;22 Suppl 4:S1–14. . 7523767 10.1097/00005344-199322004-00002

[16] Wolf HK, Zarnegar R, Michalopoulos GK. Localization of hepatocyte growth factor in human and rat tissues: an immunohistochemical study. Hepatology. 1991;14(3):488–94. Epub 1991/09/01. . 1831438

[17] Besse S, Boucher F, Linguet G, Riou L, De Leiris J, Riou B, et al Intramyocardial protein therapy with vascular endothelial growth factor (VEGF-165) induces functional angiogenesis in rat senescent myocardium. J Physiol Pharmacol. 2010;61(6):651–61. Epub 2011/01/13. . 21224495

[18] Jang HS, Kim HJ, Kim JM, Lee YS, Kim KL, Kim JA, et al A novel ex vivo angiogenesis assay based on electroporation-mediated delivery of naked plasmid DNA to skeletal muscle. Molecular therapy the journal of the American Society of Gene Therapy. 2004;9(3):464–74. doi: 10.1016/j.ymthe.2003.12.002 . 15006615 10.1016/j.ymthe.2003.12.002

[19] Hochman JS, Choo H. Limitation of myocardial infarct expansion by reperfusion independent of myocardial salvage. Circulation. 1987;75(1):299–306. . 3791612 10.1161/01.cir.75.1.299

[20] Florczyk U, Czauderna S, Stachurska A, Tertil M, Nowak W, Kozakowska M, et al Opposite effects of HIF-1alpha and HIF-2alpha on the regulation of IL-8 expression in endothelial cells. Free radical biology & medicine. 2011;51(10):1882–92. doi: 10.1016/j.freeradbiomed.2011.08.023 . 21925595 10.1016/j.freeradbiomed.2011.08.023 PMC3202637

[21] Chambers SE, O–Neill CL, O–Doherty TM, Medina RJ, Stitt AW. The role of immune-related myeloid cells in angiogenesis. Immunobiology. 2013;218(11):1370–5. doi: 10.1016/j.imbio.2013.06.010 . 23932437 10.1016/j.imbio.2013.06.010

[22] Nahrendorf M, Swirski FK, Aikawa E, Stangenberg L, Wurdinger T, Figueiredo JL, et al The healing myocardium sequentially mobilizes two monocyte subsets with divergent and complementary functions. The Journal of experimental medicine. 2007;204(12):3037–47. doi: 10.1084/jem.20070885 . 18025128 10.1084/jem.20070885 PMC2118517

[23] Loboda A, Jozkowicz A, Dulak J. HIF-1 versus HIF-2—is one more important than the other? Vascular pharmacology. 2012;56(5–6):245–51. doi: 10.1016/j.vph.2012.02.006 . 22366374 10.1016/j.vph.2012.02.006

[24] Wolff JA, Budker V. The mechanism of naked DNA uptake and expression. Adv Genet. 2005;54:3–20. doi: 10.1016/S0065-2660(05)54001-X . 16096005 10.1016/S0065-2660(05)54001-X

[25] Morishita R, Nakamura S, Hayashi S, Taniyama Y, Moriguchi A, Nagano T, et al Therapeutic angiogenesis induced by human recombinant hepatocyte growth factor in rabbit hind limb ischemia model as cytokine supplement therapy. Hypertension. 1999;33(6):1379–84. Epub 1999/06/18. . 10373220 10.1161/01.hyp.33.6.1379

[26] Ueda H, Nakamura T, Matsumoto K, Sawa Y, Matsuda H, Nakamura T. A potential cardioprotective role of hepatocyte growth factor in myocardial infarction in rats. Cardiovascular research. 2001;51(1):41–50. . 11399246 10.1016/s0008-6363(01)00272-3

[27] Salimath AS, Phelps EA, Boopathy AV, Che PL, Brown M, Garcia AJ, et al Dual delivery of hepatocyte and vascular endothelial growth factors via a protease-degradable hydrogel improves cardiac function in rats. PloS one. 2012;7(11):e50980 Epub 2012/12/12. doi: 10.1371/journal.pone.0050980 . 23226440 10.1371/journal.pone.0050980 PMC3511447

[28] Senyo SE, Steinhauser ML, Pizzimenti CL, Yang VK, Cai L, Wang M, et al Mammalian heart renewal by pre-existing cardiomyocytes. Nature. 2013;493(7432):433–6. doi: 10.1038/nature11682 . 23222518 10.1038/nature11682 PMC3548046

[29] Iwasaki M, Adachi Y, Nishiue T, Minamino K, Suzuki Y, Zhang Y, et al Hepatocyte growth factor delivered by ultrasound-mediated destruction of microbubbles induces proliferation of cardiomyocytes and amelioration of left ventricular contractile function in Doxorubicin-induced cardiomyopathy. Stem Cells. 2005;23(10):1589–97. doi: 10.1634/stemcells.2005-0049 . 16109756 10.1634/stemcells.2005-0049

[30] Gude N, Muraski J, Rubio M, Kajstura J, Schaefer E, Anversa P, et al Akt promotes increased cardiomyocyte cycling and expansion of the cardiac progenitor cell population. Circulation research. 2006;99(4):381–8. doi: 10.1161/01.RES.0000236754.21499.1c . 16840722 10.1161/01.RES.0000236754.21499.1c

[31] Tao Z, Chen B, Zhao Y, Chen H, Wang L, Yong Y, et al HGF percutaneous endocardial injection induces cardiomyocyte proliferation and rescues cardiac function in pigs. J Biomed Res. 2010;24(3):198–206. doi: 10.1016/S1674-8301(10)60029-2 . 23554631 10.1016/S1674-8301(10)60029-2 PMC3596555

[32] Gerritsen ME, Tomlinson JE, Zlot C, Ziman M, Hwang S. Using gene expression profiling to identify the molecular basis of the synergistic actions of hepatocyte growth factor and vascular endothelial growth factor in human endothelial cells. British journal of pharmacology. 2003;140(4):595–610. doi: 10.1038/sj.bjp.0705494 . 14504135 10.1038/sj.bjp.0705494 PMC1574080

[33] Xin X, Yang S, Ingle G, Zlot C, Rangell L, Kowalski J, et al Hepatocyte growth factor enhances vascular endothelial growth factor-induced angiogenesis in vitro and in vivo. The American journal of pathology. 2001;158(3):1111–20. doi: 10.1016/S0002-9440(10)64058-8 . 11238059 10.1016/S0002-9440(10)64058-8 PMC1850376

[34] Makarevich PI, Dergilev KV, Tsokolaeva ZI, Gluhanyuk EV, Parfyonova YEV. Combined gene delivery of VEGF and HGF induces angiogenesis and proliferation in ischemic rat myocardium. Eur J Heart Fail. 2013;12:S44–S.

[35] Potente M, Gerhardt H, Carmeliet P. Basic and therapeutic aspects of angiogenesis. Cell. 2011;146(6):873–87. doi: 10.1016/j.cell.2011.08.039 . 21925313 10.1016/j.cell.2011.08.039

[36] Aoki M, Morishita R, Taniyama Y, Kida I, Moriguchi A, Matsumoto K, et al Angiogenesis induced by hepatocyte growth factor in non-infarcted myocardium and infarcted myocardium: up-regulation of essential transcription factor for angiogenesis, ets. Gene therapy. 2000;7(5):417–27. Epub 2000/03/01. doi: 10.1038/sj.gt.3301104 . 10694824 10.1038/sj.gt.3301104

[37] Shirakawa Y, Sawa Y, Takewa Y, Tatsumi E, Kaneda Y, Taenaka Y, et al Gene transfection with human hepatocyte growth factor complementary DNA plasmids attenuates cardiac remodeling after acute myocardial infarction in goat hearts implanted with ventricular assist devices. The Journal of thoracic and cardiovascular surgery. 2005;130(3):624–32. doi: 10.1016/j.jtcvs.2004.02.045 . 16153905 10.1016/j.jtcvs.2004.02.045

[38] van Royen N, Piek JJ, Buschmann I, Hoefer I, Voskuil M, Schaper W. Stimulation of arteriogenesis; a new concept for the treatment of arterial occlusive disease. Cardiovascular research. 2001;49(3):543–53. . 11166267 10.1016/s0008-6363(00)00206-6

[39] Jetten N, Donners MM, Wagenaar A, Cleutjens JP, van Rooijen N, de Winther MP, et al Local delivery of polarized macrophages improves reperfusion recovery in a mouse hind limb ischemia model. PloS one. 2013;8(7):e68811 doi: 10.1371/journal.pone.0068811 . 23894348 10.1371/journal.pone.0068811 PMC3722193

[40] Hoefer IE, Grundmann S, van Royen N, Voskuil M, Schirmer SH, Ulusans S, et al Leukocyte subpopulations and arteriogenesis: specific role of monocytes, lymphocytes and granulocytes. Atherosclerosis. 2005;181(2):285–93. doi: 10.1016/j.atherosclerosis.2005.01.047 . 16039282 10.1016/j.atherosclerosis.2005.01.047

[41] Bergmann CE, Hoefer IE, Meder B, Roth H, van Royen N, Breit SM, et al Arteriogenesis depends on circulating monocytes and macrophage accumulation and is severely depressed in op/op mice. Journal of leukocyte biology. 2006;80(1):59–65. doi: 10.1189/jlb.0206087 . 16684892 10.1189/jlb.0206087

[42] Croll SD, Ransohoff RM, Cai N, Zhang Q, Martin FJ, Wei T, et al VEGF-mediated inflammation precedes angiogenesis in adult brain. Experimental neurology. 2004;187(2):388–402. doi: 10.1016/j.expneurol.2004.02.010 . 15144865 10.1016/j.expneurol.2004.02.010

[43] Kim I, Moon SO, Park SK, Chae SW, Koh GY. Angiopoietin-1 reduces VEGF-stimulated leukocyte adhesion to endothelial cells by reducing ICAM-1, VCAM-1, and E-selectin expression. Circulation research. 2001;89(6):477–9. . 11557733 10.1161/hh1801.097034

[44] Clauss M, Weich H, Breier G, Knies U, Rockl W, Waltenberger J, et al The vascular endothelial growth factor receptor Flt-1 mediates biological activities. Implications for a functional role of placenta growth factor in monocyte activation and chemotaxis. The Journal of biological chemistry. 1996;271(30):17629–34. . 8663424 10.1074/jbc.271.30.17629

[45] Barleon B, Sozzani S, Zhou D, Weich HA, Mantovani A, Marme D. Migration of human monocytes in response to vascular endothelial growth factor (VEGF) is mediated via the VEGF receptor flt-1. Blood. 1996;87(8):3336–43. . 8605350

[46] Son BR, Marquez-Curtis LA, Kucia M, Wysoczynski M, Turner AR, Ratajczak J, et al Migration of bone marrow and cord blood mesenchymal stem cells in vitro is regulated by stromal-derived factor-1-CXCR4 and hepatocyte growth factor-c-met axes and involves matrix metalloproteinases. Stem Cells. 2006;24(5):1254–64. doi: 10.1634/stemcells.2005-0271 . 16410389 10.1634/stemcells.2005-0271

[47] Kollet O, Shivtiel S, Chen YQ, Suriawinata J, Thung SN, Dabeva MD, et al HGF, SDF-1, and MMP-9 are involved in stress-induced human CD34+ stem cell recruitment to the liver. The Journal of clinical investigation. 2003;112(2):160–9. doi: 10.1172/JCI17902 . 12865405 10.1172/JCI17902 PMC164291

[48] Muller T, Bain G, Wang X, Papkoff J. Regulation of epithelial cell migration and tumor formation by beta-catenin signaling. Experimental cell research. 2002;280(1):119–33. . 12372345 10.1006/excr.2002.5630

[49] Beilmann M, Vande Woude GF, Dienes HP, Schirmacher P. Hepatocyte growth factor-stimulated invasiveness of monocytes. Blood. 2000;95(12):3964–9. . 10845935

[50] Kostner KM, Fahti RB, Case C, Hobson P, Tate J, Marwick TH. Inflammation, complement activation and endothelial function in stable and unstable coronary artery disease. Clinica chimica acta; international journal of clinical chemistry. 2006;365(1–2):129–34. doi: 10.1016/j.cca.2005.08.028 . 16236275 10.1016/j.cca.2005.08.028

[51] Kiefer F, Siekmann AF. The role of chemokines and their receptors in angiogenesis. Cellular and molecular life sciences: CMLS. 2011;68(17):2811–30. doi: 10.1007/s00018-011-0677-7 . 21479594 10.1007/s00018-011-0677-7 PMC11115067

[52] Heidemann J, Ogawa H, Dwinell MB, Rafiee P, Maaser C, Gockel HR, et al Angiogenic effects of interleukin 8 (CXCL8) in human intestinal microvascular endothelial cells are mediated by CXCR2. The Journal of biological chemistry. 2003;278(10):8508–15. doi: 10.1074/jbc.M208231200 . 12496258 10.1074/jbc.M208231200

[53] Shireman PK. The chemokine system in arteriogenesis and hind limb ischemia. Journal of vascular surgery: official publication, the Society for Vascular Surgery [and] International Society for Cardiovascular Surgery, North American Chapter. 2007;45 Suppl A:A48–56. . 17544024 10.1016/j.jvs.2007.02.030 PMC2680944

[54] Kunsch C, Rosen CA. NF-kappa B subunit-specific regulation of the interleukin-8 promoter. Molecular and cellular biology. 1993;13(10):6137–46. . 8413215 10.1128/mcb.13.10.6137-6146.1993 PMC364673

[55] Minet E, Arnould T, Michel G, Roland I, Mottet D, Raes M, et al ERK activation upon hypoxia: involvement in HIF-1 activation. FEBS letters. 2000;468(1):53–8. . 10683440 10.1016/s0014-5793(00)01181-9

[56] Kojima I, Tanaka T, Inagi R, Nishi H, Aburatani H, Kato H, et al Metallothionein is upregulated by hypoxia and stabilizes hypoxia-inducible factor in the kidney. Kidney international. 2009;75(3):268–77. doi: 10.1038/ki.2008.488 . 19148152 10.1038/ki.2008.488

[57] Li A, Varney ML, Valasek J, Godfrey M, Dave BJ, Singh RK. Autocrine role of interleukin-8 in induction of endothelial cell proliferation, survival, migration and MMP-2 production and angiogenesis. Angiogenesis. 2005;8(1):63–71. doi: 10.1007/s10456-005-5208-4 . 16132619 10.1007/s10456-005-5208-4

[58] Fong GH. Regulation of angiogenesis by oxygen sensing mechanisms. J Mol Med (Berl). 2009;87(6):549–60. Epub 2009/03/17. doi: 10.1007/s00109-009-0458-z . 19288062 10.1007/s00109-009-0458-z

[59] Sulpice E, Ding S, Muscatelli-Groux B, Berge M, Han ZC, Plouet J, et al Cross-talk between the VEGF-A and HGF signalling pathways in endothelial cells. Biol Cell. 2009;101(9):525–39. doi: 10.1042/BC20080221 . 19281453 10.1042/BC20080221

