# Supplementary figures and images for: Angiogenic and pleiotropic effects of VEGF165 and HGF combined gene therapy in a rat model of myocardial infarction
Source: PLoS One. 2018 May 22;13(5):e0197566. doi: 10.1371/journal.pone.0197566 (PMC5963747; doi:10.1371/journal.pone.0197566)

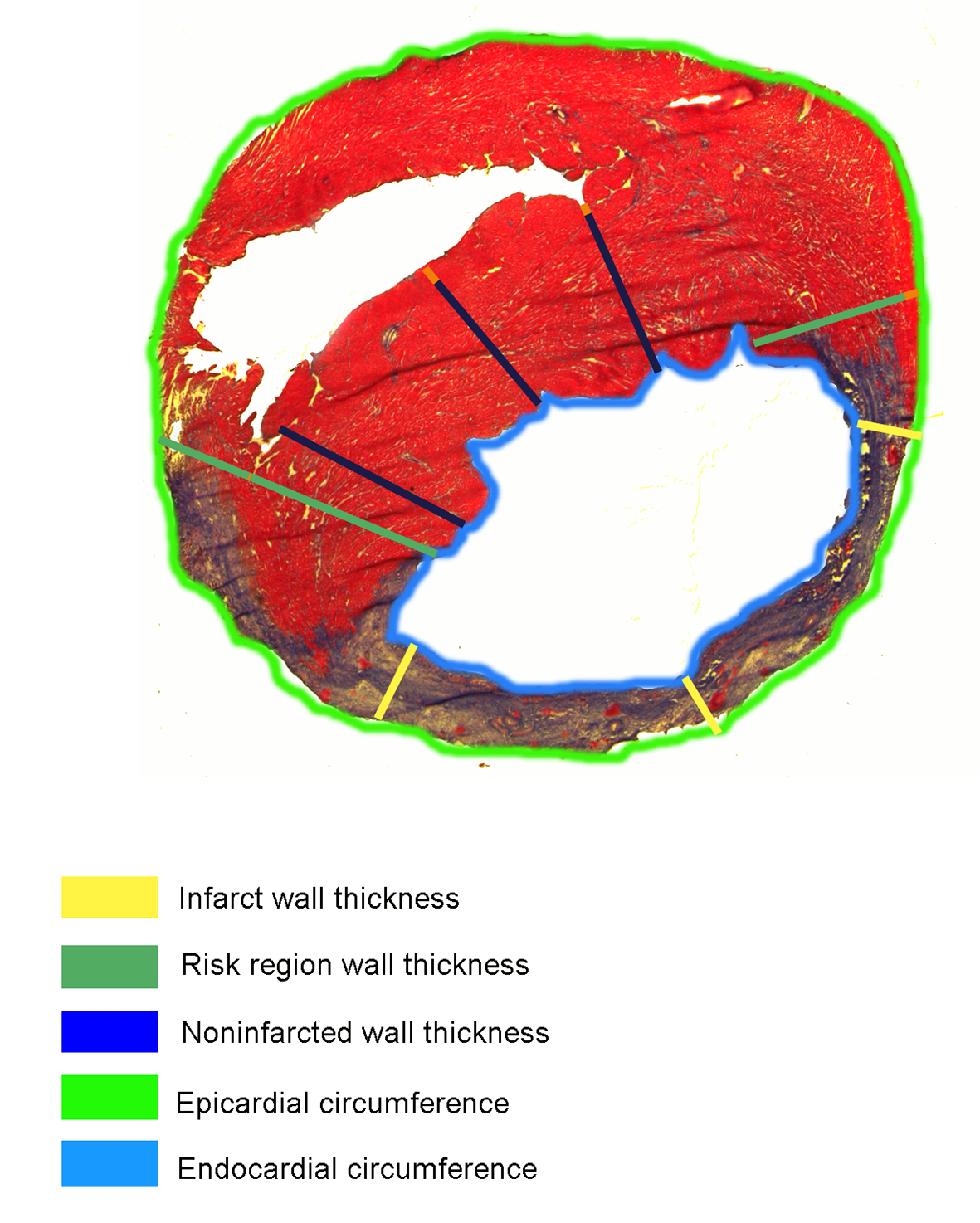

Supplement: S1 Fig — (TIF) [file pone.0197566.s001.tif]

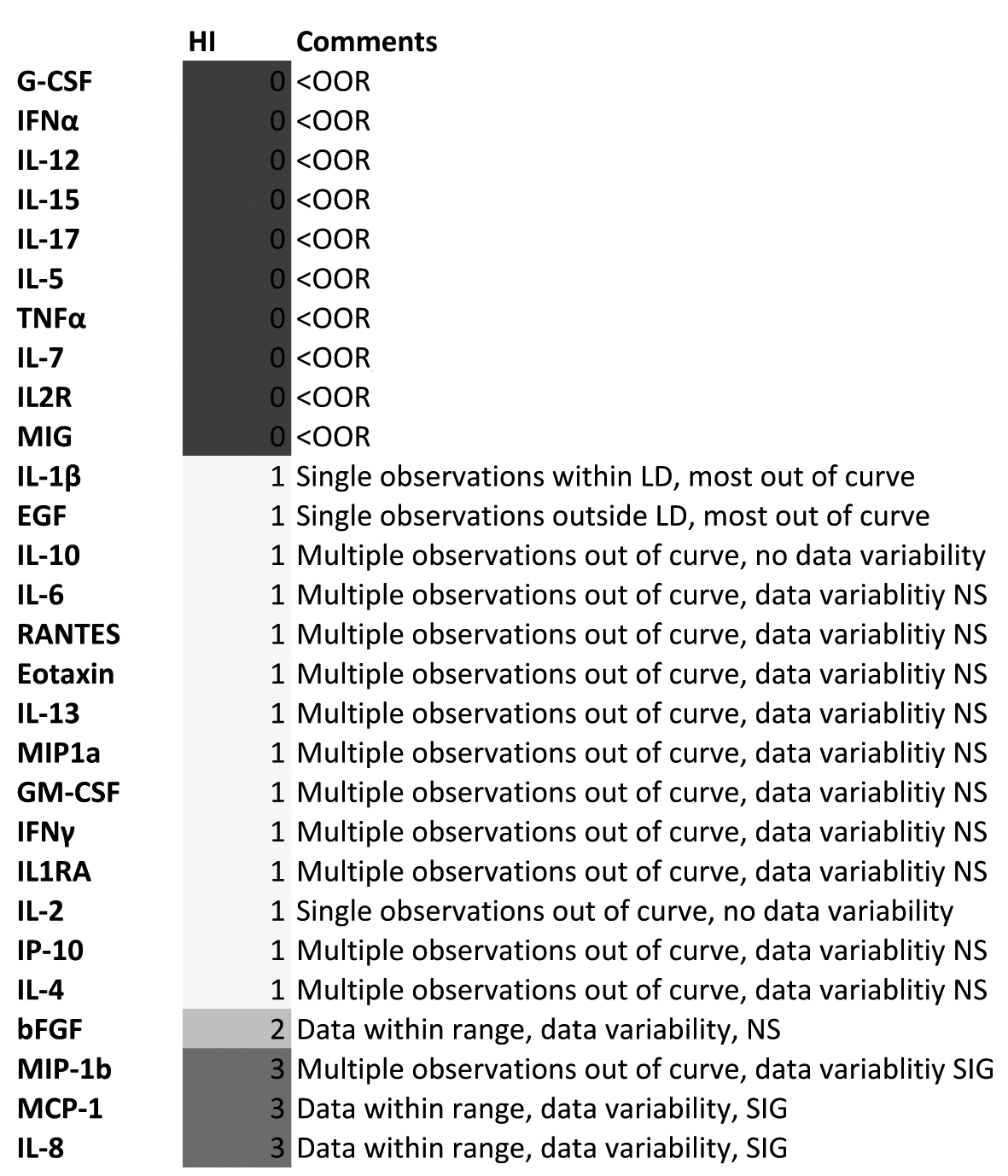

Supplement: S2 Fig — HI—heat index, OOR—our of range, LD- limit of detection, NS—not significant difference between samples, SIG—significant difference between samples. (TIF) [file pone.0197566.s002.tif]
