# Supplementary material for: Angiogenic and pleiotropic effects of VEGF165 and HGF combined gene therapy in a rat model of myocardial infarction
Source: PLoS One. 2018 May 22;13(5):e0197566. doi: 10.1371/journal.pone.0197566 (PMC5963747; doi:10.1371/journal.pone.0197566)
Supplement: S1 Table — (DOCX) [file pone.0197566.s003.docx]

**S1 Table. Primers used for semi-quantitative PCR**

| **cDNA** | **Sense primer** | **Anti-sense primer** |
| --- | --- | --- |
| \| **ACTB** \| 5’-cctggcacccagcacaat-3’ \| 5’-gggccggactcgtcatac-3’ \| \| --- \| --- \| --- \| \| **GAPDH** \| 5’-tgcaccaccaactgcttagc-3’ \| 5’-ggcatggactgtggtcatgag-3’ \| \| **IL-8** \| 5’-ctggccgtggctctcttg-3’ \| 5’-ccttggcaaaactgcacctt-3’ \| \| **MCP-1** \| 5’- cagccagatgcaatcaatgc-3’ \| 5’- gcactgagatcttcctattggtgaa-3’ \| \| **HIF-1α** \| 5’-gcaagccctgaaagcg-3’ \| 5’-ggctgtccgactttga-3’ \| \| **HIF-2α** \| 5’-gtctctccaccccatgtctc-3’ \| 5’-ggttcttcatccgtttccac-3’ \| | | |
